# The effects of acupuncture on pregnancy outcomes of in vitro fertilization: a systematic review and meta-analysis

**DOI:** 10.1186/s12906-019-2523-7

**Published:** 2019-06-14

**Authors:** Zheng-yun Xie, Zhi-hang Peng, Bing Yao, Li Chen, Yan-yun Mu, Jie Cheng, Qian Li, Xi Luo, Peng-yan Yang, You-bing Xia

**Affiliations:** 1 Nanjing Hospital of Traditional Chinese Medicine, The Third Affiliated Hospital of Nanjing University of Chinese Medicine, Nanjing, China; 2 Department of Epidemiology and Biostatistics, School of Public Health, Nanjing Medical University, Nanjing, China; 3 Center of Reproductive Medicine, Nanjing Jinling Hospital, Nanjing University School of Medical, Nanjing, China; 4 The Second School Medical College, Nanjing University of Chinese Medicine, Nanjing, China; 5 Changzheng Hospital of the Second Military Medical University, Nanjing branch, Nanjing, China; 6 Nanjing Jiangning Hospital of Traditional Chinese Medicine, Nanjing, China; 7 Xuzhou Medical University, Xuzhou, China

**Keywords:** Acupuncture, In vitro fertilization, Clinical pregnancy rate, Live birth rate, Systematic review

## Abstract

**Background:**

The effects of acupuncture on in vitro fertilization (IVF) outcomes remain controversial. And the variation in participant, interventions, outcomes studied, and trial design may relate to the efficacy of adjuvant acupuncture.

**Methods:**

We searched digital databases for relevant studies, including Embase, PubMed, Cochrane Library and some Chinese databases up to December 2018, for randomized controlled trials (RCTs) evaluating the effects of acupuncture on women undergoing IVF. We included studies with intervention groups using needling, and control groups consisting of no acupuncture or sham (placebo) acupuncture. Primary outcomes were clinical pregnancy rate (CPR) and live birth rate (LBR). Meta-regression and subgroup analysis were conducted on the basis of eight pre-specified covariates to investigate the variances of the effects of adjuvant acupuncture on pregnancy rates and the sources of heterogeneity.

**Results:**

Twenty-seven studies with 6116 participants were included. The pooled clinical pregnancy rate (CPR) from all of acupuncture groups was significantly greater than that of control groups (RR 1.21, 95% CI: 1.07–1.38), whereas the pooled live birth rate (LBR) was not. Meta-regression subgroup analysis showed a more significant benefit of acupuncture for repeated IVF cycle proportion (number of women with a history of prior unsuccessful IVF attempt divided by number of women included in each trial) ≥ 50% group (CPR: RR 1.60, 95% CI: 1.28–2.00; LBR: RR 1.42, 95% CI: 1.05–1.92), and this covariate explained most of the heterogeneity (CPR and LBR: adjusted *R*^*2*^ = 100 and 87.90%). Similar results were found between CPR and number of acupuncture treatments (CPR: *p* = 0.002, adjusted *R*^*2*^ = 51.90%), but not LBR.

**Conclusions:**

Our analysis finds a benefit of acupuncture for IVF outcomes in women with a history of unsuccessful IVF attempt, and number of acupuncture treatments is a potential influential factor. Given the poor reporting and methodological flaws of existing studies, studies with larger scales and better methodologies are needed to verify these findings.

**Electronic supplementary material:**

The online version of this article (10.1186/s12906-019-2523-7) contains supplementary material, which is available to authorized users.

## Introduction

12.5% women failure to establish a clinical pregnancy after 12 months of regular, unprotected sexual intercourse [1, 2]. One of the most successful and commonly utilized treatment options is IVF and its related derivative technologies. In 2011 worldwide, approximately 2.0 million IVF cycles were reported, resulting in 0.5 million babies born [3]. Over the past few decades, technological advances in IVF, including advances in protocols for ovarian stimulation, oocyte retrieval, fertilization, and embryo culture and transfer, have resulted in more efficient approaches to treating infertility [4]. Nevertheless, delivery rates (DR) per aspiration remain low, with rates of 19.8% in 2011 worldwide [3], and 22.3% in Europe, 2013 [5]. For many women, cycles need to be repeated to be successful. Moreover, the IVF cycle is time-consuming and costly, including controlled ovarian hyperstimulation (COH), oocyte retrieval, IVF/intracytoplasmic sperm injection (ICSI), embryo transfer (ET) and luteal support, with costs range from $15,000 to $18,000 per cycle, including medications. And the preimplantation genetic diagnosis (PGD) step of the process meant another $3000 to $6000. Altogether, conservatively speaking, about $20,000 [6–9]. Methods that are effective in improving IVF outcomes may reduce the need for an additional high-cost IVF cycle. Therefore, there is a need to explore new techniques and therapies to improve the success rates of IVF.

Acupuncture and other modalities of Chinese/East Asian medicine have been used in women’s health for many centuries. They are safe in the hands of competent practitioners [10, 11], even for pregnant women suffering various complications (e.g. hyperemesis gravidarum) [12, 13]. Many studies have reported that acupuncture improved pregnancy rates among women undergoing IVF [14–22], which may relate to the following mechanisms, including regulating the function of the hypothalamic–pituitary–ovarian axis by changing the concentration of central opioids [23–26], improving blood circulation to the uterus and ovaries by inhibiting uterine central sympathetic nerve activity [17, 27] and reducing stress, anxiety or depression [28–30]. Subsequently, many systematic reviews analyzed the effects of acupuncture among women undergoing IVF [31–43], however, these meta-analyses have led to different conclusions. Potential reasons for these discrepancies include differences in how studies are selected, and how participants, interventions and outcomes are defined by reviewers [44]. Consequently, it may be difficult to draw definitive conclusions based on previous meta-analyses. Hence, we conducted a new systematic review and meta-analysis of randomized controlled trials (RCTs) involving previously defined set of subgroup analyses and meta-regressions to explore whether variation among participants, interventions, outcomes studied, and trial design influence estimates of the effects of adjuvant acupuncture on pregnancy outcomes in women undergoing IVF.

## Methods

### Search strategy

We searched digital databases for relevant studies, including Embase, PubMed, Cochrane Library and some Chinese databases, such as WanFang, CNKI, VIP and the Chinese SinoMed Database (up to December 2018). The MEDLINE search strategy is given in Additional file 4: Table S1.

The following were used as free text terms and MeSH terms: acupuncture therapy; acupuncture; electroacupuncture; auriculotherapy; auricular acupuncture; acupuncture analgesia; acup* and reproductive techniques, assisted; assisted reproducti*; in vitro fertili*; intracytoplasmic sperm injection; embryo transfer; embryo implantation; oocyte; egg collection. We combined this search strategy with a filter for clinical trials.

The following terms were used in the Chinese database searches: “ZHEN JIU” (which means “acupuncture and moxibustion”); “ZHEN CI” (which means “acupuncture”); “DIAN ZHEN” (which means “electroacupuncture”); “ER ZHEN” (which means “auricular acupuncture”) and “FU ZHU SHEN ZHI” (which means “assisted reproductive technology”); “TI WAI SHOU JING” (which means “*in vitro* fertilization”); “SHI GUAN YING ER” (which means “test tube baby”); “LUAN BAO JIANG NEI DAN JING ZI XIAN WEI ZHU SHE” (which means “intracytoplasmic sperm injection”); PEI TAI YI ZHI (which means “embryo transfer”).

We also scanned the Index to Scientific & Technical Proceedings (Web of Science), and the reference lists of relevant primary and review articles were examined to identify cited articles not captured by electronic searches.

### Study selection

The inclusion criteria were as follows: (1) RCTs that evaluated the effects of acupuncture on IVF outcomes in women undergoing IVF, with or without ICSI. Namely, women in intervention groups received both IVF and acupuncture and control groups received IVF with or without sham/placebo acupuncture; (2) no restriction on objective of study, meaning we included studies where acupuncture was administered for pain relief during oocyte retrieval, or for anxiety relief during IVF-ET, or for improving IVF outcomes; (3) any of three types of acupuncture: manual (MA), electrical (EA), and auricular acupuncture techniques; (4) studies using either traditional acupuncture, in which needles were inserted in classical meridian points, or western medical acupuncture [45], in which the needles were inserted in non-meridian or trigger points; (5)a clear description of acupuncture time. That is, we included studies which give an equal number of acupuncture treatments for women within a group; (6) needling in the control groups could be either no acupuncture or sham (placebo) acupuncture; (7) both fresh and frozen–thawed embryo transfer cycles reporting at least one of the following outcomes: clinical pregnancy rates (CPR - a pregnancy diagnosed by ultrasonographic visualization of one or more gestational sacs or definitive clinical signs of pregnancy), or live birth rates (LBR - the complete expulsion or extraction from a woman of a product of fertilization, after 22 completed weeks of gestational age; which, after such separation, breathes or shows any other evidence of life, such as heart beat, umbilical cord pulsation or definite movement of voluntary muscles, irrespective of whether the umbilical cord has been cut or the placenta is attached) [2]; and (8) no restrictions on publication type or language. Where studies had multiple publications, the main trial report was used as the reference and additional details were derived from secondary papers.

Exclusion criteria were as follows: studies of acupuncture treatments without needling; retrospective studies, case series, and studies with a crossover design.

### Data extraction

Specific characteristics were extracted from each study: first author, year, demographic characteristics (i.e. age, IVF cycle number, duration of infertility, type of infertility, number of embryos transferred), invention (i.e. acupuncture type, acupoints, acupuncture time, number of acupuncture treatments), type of control, IVF outcomes and methodological quality of the trials.

Study selection and data extraction were completed in duplicate and independently by two investigators (ZYX and XL). A third reviewer (ZHP) independently assessed the study for consensus in case of disagreement. We corresponded with study investigators to clarify further data on methods and results. The relevant primary and review articles were also examined to identify further data on methods and results not published in the papers.

### Quality assessment

Included studies were assessed for risk of bias using the Cochrane risk of bias assessment tool [46] to assess the following domains: random sequence generation; allocation concealment; blinding of patients; incomplete outcome data; selective reporting; and other bias. The GRADE methodology was used to assess the quality of retrieved evidence (GRADEpro, Version 3.6 for Windows, Grade Working group). The level of evidence was categorized into 4 levels: high, moderate, low, or very low quality.

### Outcome measures

We pre-specified clinical pregnancy rate (CPR) and live birth rate (LBR) as our primary outcomes.

Miscarriage rate (MR: [CPR-LBR]/CPR) and any reported side effects from the treatments were analyzed as secondary outcomes.

### Statistical analysis

Data were analyzed in accordance with the Cochrane Handbook for Systematic Reviews of Interventions [47]. All data were dichotomous. Results were pooled and expressed as relative risks (RR) with 95% confidence intervals (CI) using RevMan V.5.3 meta-analysis software (The Nordic Cochrane Centre, The Cochrane Collaboration, 2014). Because of the expected heterogeneity of acupuncture protocols, controls, and demographic characteristics, a random effects model was used. Heterogeneity of treatment effects was evaluated graphically using Forest plot, and statistically by *I*^*2*^ statistic and chi-square test. We performed both an available case analysis for a main analysis and an intention-to-treat analysis (ITT) for sensitivity analysis, which means all of the meta-analyses were based on both the number of whose results are known (available case analysis) and the number of women randomized (ITT).

### Subgroup analyses

We conducted subgroup analyses on the basis of the following covariates: (1) age (< 33.3 or ≥ 33.3 years); (2) duration of infertility (< 5.6 or ≥ 5.6 years). Mean age was 33.3 years and duration of infertility was 5.6 years for the largest IVF success rates prediction study (144,018 cycles) which assessed the extent to which baseline characteristics be predict pregnancy rates [48]; (3) type of infertility (primary infertility proportion < 50% or ≥ 50%); (4) repeated IVF cycle proportion (number of women with a history of prior unsuccessful IVF attempt divided by number of women included in each trial) < 50% or ≥ 50%; (5) number of embryos transferred (< 1.9 or ≥ 1.9, which was the average number of embryos transferred in 2011 globally [3]); (6) number of acupuncture treatments (one or more than one session). We classified protocols in which acupuncture was performed 25 min prior to and after ET as one session, others counted as descriptions in each study. This is because embryo transfer requires only a few minutes, and the interval of two acupuncture treatments performed 25 min before and after ET is very short. Considering that the physiological effects of acupuncture can continue for many hours [49, 50], it is reasonable to classify this protocol as one acupuncture treatment; (7) acupuncture type (electroacupuncture or manual acupuncture); (8) type of control (no or sham (placebo) acupuncture invention control).

Subsequently, a random effects meta-regression analysis was conducted for each variable in STATA version 14.0 (Stata Corporation, College Station, TX, USA). *P*-value was significant at the 0.05 level. Each variable tested independently in the model. Considering meta-analysis allows for residual heterogeneity among intervention effects not modelled by the explanatory variables, it is reasonable to use a random effects meta-regression analysis [51]. Due to some variables in our meta-analysis being mean values, we also considered the existence of aggregation bias (also known as ecological bias or the ecological fallacy) [52–54]. Multivariate meta-regression was not conducted in our meta-analysis primarily because of the limited number of included studies for each subgroup and our objective was to identify the relevant influential factors for the effects of acupuncture on IVF outcomes.

### Publication bias

Publication bias was visually evaluated using funnel plots when at least 10 trials were available for meta-analysis. Subsequently, Egger’s test [55] was performed to statistically assess the degree of asymmetry, and a cumulative meta-analysis was conducted to identify the potential for small-study effects. A two-tailed *p* value < 0.05 was considered statistically significant.

### Sensitivity analyses

The sensitivity analyses were performed to explore whether the overall conclusions were affected under the four scenarios: (1) if we included randomized participants whose pregnancy outcome data were missing; (2) if we re-classified before and after ET protocols into two acupuncture sessions; (3) if we restricted CPR results to the 15 studies which reported LBR; and (4) if we performed the meta-regression and subgroup analyses stratified by control type.

In addition, we performed the meta-regression subgroup analyses according to ‘risk of bias’ of included studies.

## Results

The selection process is documented in a PRISMA flow chart in Additional file 1: Figure S1. Reasons for excluding studies are given in Additional file 8: Appendix 1. Twenty-seven randomized controlled trials [14–22, 29, 56–72] with a total of 6116 participants met inclusion criteria. The characteristics of the included studies are displayed in Table 1.

**Table 1:** Characteristics of the studies included in this review

Study	Country	Main objective	Acupuncture location	No. randomised	Acupuncture sessions	Acupuncture type	De qi sensation	Control^a^	IVF outcomes
Andersen 2010 [65]	Denmark	IVF outcome	On-site	635	Two sessions: (1) 30 m before ET; (2) immediately after ET (*n* = 314)	MA	Yes	The Streitberger placebo needles^b^ placed on the same acupoints which the true acupuncture used (*n* = 321)	BPR、CPR、OPR、LBR、MR、IR
Arnoldi 2010 [18]	Italy	IVF outcome	On-site	204	Three sessions: (1) d 5 of ovarian stimulation; (2) 30 m before ET; (3) immediately after ET (*n* = 102)	MA	Yes	No adjuvant treatment (n = 102)	CPR、LBR、MR、IR
Craig 2014 [59]	USA	IVF outcome	Off-site	113	Two sessions: (1) within 1–2 h before ET; (2) within 1–2 h after ET (*n* = 57)	MA	Yes	No adjuvant treatment (*n* = 56)	BPR、CPR、LBR、MR
Dieterle 2006 [15]	Germany	IVF outcome	On-site	225	Two sessions: (1) immediately after ET; (2) 3 days after ET (*n* = 116)	MA	Yes	Needles inserted in real acupoints which were designed not to influence fertility (*n* = 109)	BPR、CPR、OPR、LBR、MR、IR
Domar 2009 [29]	USA	IVF outcome	On-site	150	Two sessions: (1) 25 m before ET; (2) immediately after ET (*n* = 78)^d^	MA	Unclear	No adjuvant treatment (*n* = 68)^d^	BPR、CPR
Feliciani 2011 [62]	Italy	IVF outcome	On-site	46	Three sessions: (1) 5–7d before OA; (2) 2–3 d before OA; (3) within 1 h after ET (n = 23)	MA	Yes	No adjuvant treatment (*n* = 23)	CPR、OPR、LBR、MR、IR
Gillerman 2016 [58]	UK	IVF outcome	On-site	127	Three sessions: (1) between days 6 and 8 of ovarian stimulation; (2) before egg retrieval; (3) before and after embryo transfer (*n* = 67)	MA	Yes	No adjuvant treatment (*n* = 60)	CPR
Ho 2009 [17]	Taiwan	IVF outcome	On-site	56	Four sessions: twice a week for 2 weeks, from d2 to the day before oocyte retrieval (*n* = 30)	EA	Yes	No adjuvant treatment (*n* = 26)^j^	CPR
Humaidan 2004 [69]	Denmark	Pain-relieving effect	On-site	200	One session: a few minutes before OA and terminated directly after OA (*n* = 75)	EA	No	Alfentanil + PCB (n = 100)	BPR、CPR
Villahermosa 2012 [20]	Brazil	anxiety-relieving efffects	On-site	43	Four sessions: once a week over a 4-week period, comprised the whole IVF process from induction of ovulation to the result of β-human chorionic gonadotrophin, the last treatment was given after embryo transfer (*n* = 22)	MA	Yes	Needles inserted in the sites which close to but not on (n = 21)	CPR
Villahermosa 2013 [21]	Brazil	IVF outcome	On-site	84	Four sessions: (1)D1 (first day of ovulation induction); (2)D7 (seventh day of ovulation induction); (3)on the day before the day of ovarian puncture; (4)on the day after the day of embryo transfer (*n* = 28)	MA	Yes	Needles inserted in the arm and thigh, which are known not to correspond with classically described acupuncture points (*n* = 46)	BPR、CPR
Madaschi 2010 [64]	Brazil	IVF outcome	On-site	455^g^	Two sessions: (1) 25 m before ET; (2) immediately after ET (*n* = 230)	MA	Yes	No adjuvant treatment (*n* = 225)	CPR、LBR、MR、IR
Morin 2017 [59]	USA	IVF outcome	On-site	424	Two sessions: (1) 25 m before ET; (2) after ET (*n* = 210)^e^	MA	Unclear	No adjuvant treatment (n = 210)^f^	BPR、CPR、OPR、LBR、MR、IR
Moy 2011 [61]	USA	IVF outcome	On-site	161	Two sessions: (1) 25 m before ET; (2) immediately after ET (*n* = 87)	MA	Yes	Needles inserted in the sites which close to, but not on, the real acupoints (*n* = 74)	BPR、CPR
Omodei 2010 [19]	Italy	IVF outcome	On-site	168^h^	Two sessions: (1) 25 m before ET; (2) immediately after ET (n = 44)^h^	MA	Unclear	No adjuvant treatment (*n* = 124)^h^	CPR、OPR、LBR、MR
Paulus 2002 [14]	Germany	IVF outcome	On-site	160	Two sessions: (1) 25 m before ET; (2) immediately after ET (*n* = 80)	MA	Yes	No adjuvant treatment (n = 80)	CPR、OPR、LBR、MR
Paulus 2003 [71]	Germany	IVF outcome	On-site	200	Two sessions: (1) 25 m before ET; (2) immediately after ET (n = 100)	MA	Yes	The Streitberger placebo needles^b^ placed on the same acupoints which the true acupuncture used (*n* = 100)	CPR、OPR、LBR、MR
Peyvandi 2016 [22]	Iran	IVF and FET outcomes	On-site	164	Three sessions: (1)oocyte puncture day; (2)2 days after puncture; (3)one cycle before embryo transfer (*n* = 82)	EA	No	No adjuvant treatment (n = 82)	BPR、CPR、OPR
Rashidi 2013 [60]	Iran	IVF outcome	On-site	62	Five sessions: (1)30 m on the 21st day of the previous cycle (start of downregulation); (2) the first day of stimulation; (3)2 days before OPU; (4) 30 m before ET; (5) immediately after ET (n = 31)	EA	Yes	No adjuvant treatment (n = 31)	BPR、CPR、OPR
Sator-K 2006 [68]	Australia	Pain-relieving effect	On-site	94	One session:30 m before OA and lasted until 1 h after OA (*n* = 64)^i^	EA/MA	No	PCA + an adhesive tape over the whole ear connected to the P-Stim™ without electrical stimulation (n = 30)	CPR
Smith 2006 [67]	Australia	IVF outcome	On-site	228	Three sessions: (1) d9 of stimulating injections; (2)25 m before ET; (3) immediately after ET (*n* = 110)	MA	Yes	The Streitberger placebo needles^b^ placed close to but not on the real acupuncture points (*n* = 118)	CPR、OPR
Smith 2018 [56]	Australia and New Zealand	IVF outcome	On-site at the IVF centers or nearby	848	Three sessions: (1) between d6 and d8 of ovarian stimulation; (2) 1 h before ET; (3) immediately after ET (*n* = 424)^k^	MA	Yes	Park sham needles^c^ placed away from known acupuncture points (n = 424)^k^	
So 2009 [66]	Hong Kong	IVF outcome	On-site	370	Two sessions: (1) 25 m before ET; (2) immediately after ET (*n* = 185)	MA	Yes	The Streitberger placebo needles^b^ placed on the same acupoints which the true acupuncture used to give patients a pricking, penetrating sensation (n = 185)	BPR、CPR、OPR、LBR、MR、IR
So 2010 [63]	Hong Kong	FET outcome	On-site	226	One session: immediately after ET (*n* = 113)	MA	Yes	The Streitberger placebo needles^b^ placed on the same acupoints which the true acupuncture used to give patients a pricking, penetrating sensation (n = 113)	BPR、CPR、OPR、LBR、MR、IR
Stener-Victorin 1999 [72]	Sweden	Pain-relieving effect	On-site	150	One session: 30 m before OA and terminated directly after OA (n = 75)	EA	Yes	Alfentanil + PCB (n = 75)	CPR、LBR、MR、IR
Stener-Victorin 2003 [70]	Sweden	IVF outcome	On-site	286	One session: 30 m before OA and terminated directly after OA (*n* = 141)	EA	Yes	Alfentanil + PCB (*n* = 145)	CPR、OPR、IR
Westergaard 2006 [16]	Denmark	IVF outcome	On-site	300	Acupuncture group 1:Two sessions: (1)25 m before ET; (2)immediatelyafter ET (n = 100); Acupuncture group 2: Three sessions: (1) 25 m before ET; (2)immediately after ET; (3) 2 d after ET (n = 100)^i^	MA	Yes	No adjuvant treatment (n = 100)	BPR、CPR、OPR、LBR、MR、IR

Note:*Acupoints* Acupuncture points, *OA* oocyte aspiration, *ET* embryo transfer, *COH* controlled ovarian hyperstimulation, *MA* manual acupuncture, *EA* electro acupuncture, *d* day, *h* hour, *m* minute, *BPR* biochemical pregnancy rate, *CPR* clinical pregnancy rate, *OPR* ongoing pregnancy rate, *LBR* live birth rate, *MR* miscarriage rate

^a^For all sham-controlled trials, the sham acupuncture procedure was given on the same schedule as that used for the true acupuncture group

^b^The tip of the Streitberger placebo needle is blunted, skin penetration did not occur

^c^Park sham needle has a retractable needle shaft and a blunt tip

^d^The treatment assignment and the outcomes for 4/150 randomized participants were not recorded by the trial authors

^e^There are two invention groups in the trial:needle acupuncture (AC) and laser acupuncture (LZ AC),and our meta-analysis only included the AC group

^f^There are three control groups in the trial:sham laser acupuncture (LZ sham), relaxation (RX) and no treatment (NT),and our meta-analysis only included the NT group

^**g**^The 39 participants (22 acupuncture; 17 control) who did not proceed to embryo transfer were excluded from the trial authors’ analysis (Manheimer, et al., 2013). Our meta-analysis was to re-include these participants and assumed the these participants failure to achieve the clinical pregnancy outcome

^h^The number of each group were not available from the publishing information, but other review reported it (Manheimer, et al., 2013)

^i^There are two intervention arms in the trial, and we combined them together for the meta-analysis

^j^8/26 patients in the control group excluded from the study (cancel to participate further after randomization)

^k^9/424 in acupuncture group and 15/424 in control group excluded from analysis (withdrawal consent for data to be used)

### Inclusion criteria

The differences among inclusion criteria for 27 studies are as follows:

Two German trials [14, 71] included only women with good-quality embryos, and one trial [21] restricted eligibility to women who had at least two previous unsuccessful attempts of IVF. The other trials included women with varying quality of embryos and number of previous IVF attempts. One trial [18] restricted eligibility to women with unfavorable reproductive prognoses, and others included women without limit to reproductive prognoses or ovarian responses. One trial [60] included participants who were infertile with polycystic ovary syndrome (PCOS) and candidates for IVF/ICSI, others included women with various causes of infertility. One trial [63] restricted eligibility to frozen–thawed embryo transfer cycles, and one [22] included both frozen–thawed and fresh embryos, while the others used fresh embryos. One trial [64] used ICSI for all participants, whereas others used of ICSI for only some participants.

### Acupuncture interventions

The timing of the acupuncture sessions differed somewhat among trials. Four studies [68–70, 72] performed one acupuncture session during oocyte aspiration (OA). Eleven studies [14, 19, 29, 57, 59, 61, 63–71] performed one or two acupuncture sessions around embryo transfer. Twelve studies performed two or more than two acupuncture sessions. Among these, ten [17, 18, 20–22, 56, 58, 60, 62, 67] performed during COH with or without ET day, two [15, 16] performed around and 2–3 days after ET.

Seven studies [17, 22, 60, 68–70, 72] used EA, and the other 21 trials used manual acupuncture (MA) as adjunctive treatment. Six trials [19, 22, 29, 57, 68, 69] did not report whether the needles used in the true acupuncture group were manipulated to achieve the de qi sensation (i.e. a sensation perceived by the patients, which manifests as numbness, heaviness, distention, and soreness, with spreading sensation and it is also perceived by the acupuncturists, which manifests as heavy and tight sensation coming from beneath the needle [73].) The remaining 21 trials needles were manually stimulated to obtain de qi sensation. Both Sator-Katzenschlager et al. [68] and Westergaard et al. [16] had two intervention groups, the former using auricular electro-acupuncture and auricular acupuncture, and the latter performed MA in different times between two invention groups. One trial [59] used off-site acupuncture, which means only patients in the acupuncture group were required to drive to and from the off-site acupuncturist’s office both before and after the embryo transfer procedure, one [56] adopted both on-site and off-site acupuncture, and the remaining 25 trials were all on-site acupuncture.

### Controls

The control groups included no acupuncture and sham (placebo) acupuncture (Table 1). Eleven trials used sham or placebo acupuncture [15, 20, 21, 56, 61, 63, 65–68, 71], the others used no acupuncture intervention as the control group. One [21] had two control groups, one sham acupuncture group and one no acupuncture control group, and these were grouped separately for relevant subgroup analyses.

### IVF outcomes

Clinical pregnancy rates were available from all the 27 trials. Fifteen [14–16, 18, 19, 56, 57, 59, 62–66, 71, 72] reported live birth rates and miscarriage rates.

### Methodological quality of the studies

**Fig. 1:**
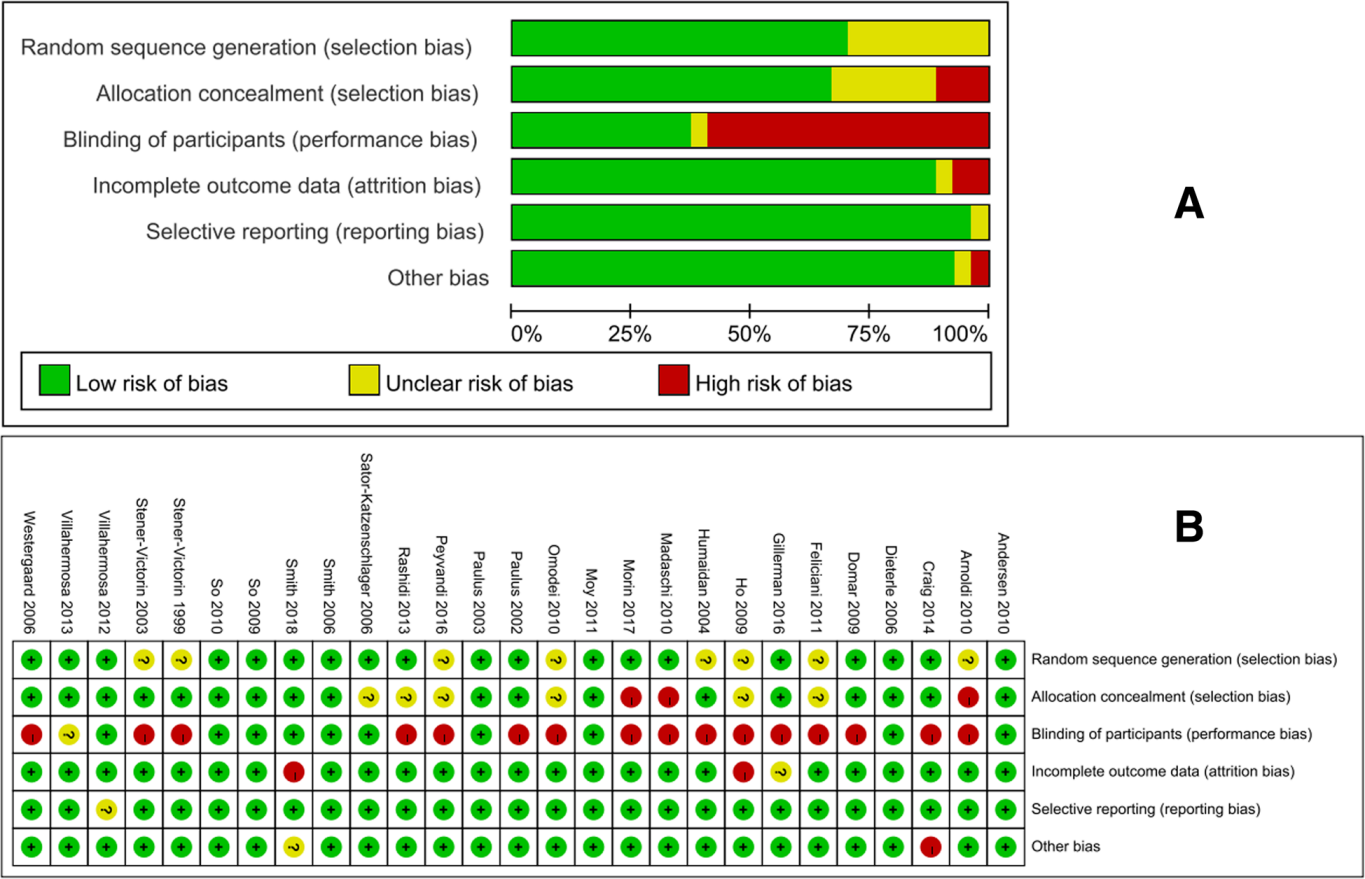
Risk of bias graph (**a**) and risk of bias summary (**b**) based on review authors’ judgments about each risk of bias item

A summary of the risks of bias in included studies is presented in Fig. 1. Nineteen studies were at low risk of selection bias related to the random sequence generation. Eight studies [17–19, 22, 62, 69, 70, 72] were at unclear randomizations as they did not describe the randomization method used. For three studies [18, 57, 64], there was inadequate allocation concealment, which placed them at high risk of bias. Six studies did not describe allocation concealment and were at unclear risk of this bias [17, 19, 22, 60, 62, 68]. However, selection bias does not appear to be an issue in these trials, because of the similar baseline among groups. Owing to the nature of acupuncture studies, absolute double blinding was often not possible. Ten trials [15, 20, 56, 61, 63, 65–68, 71] were at low risk of performance bias due to the use of sham or placebo acupuncture controls for blinding. Villahermosa et al. 2013 [21] had two control groups, a sham acupuncture group and a no acupuncture intervention control group, so we scored this criterion as “unclear risk” for blinding of patients. The remaining trials used no acupuncture intervention as the control group and were considered to be at high risk of bias. In nine trials [16, 17, 56, 59, 60, 64, 67, 69, 70], some randomized women began the IVF process but did not complete embryo transfer, nevertheless, we included these women in the meta-analysis. In ten trials [16, 17, 29, 56, 58, 59, 61, 68, 70, 72], there were small numbers of randomized women with missing clinical pregnancy outcomes, however, we re-included them in the sensitivity analysis to explore its effect size on pregnancy outcomes.

### Effects of interventions

**Fig. 2:**
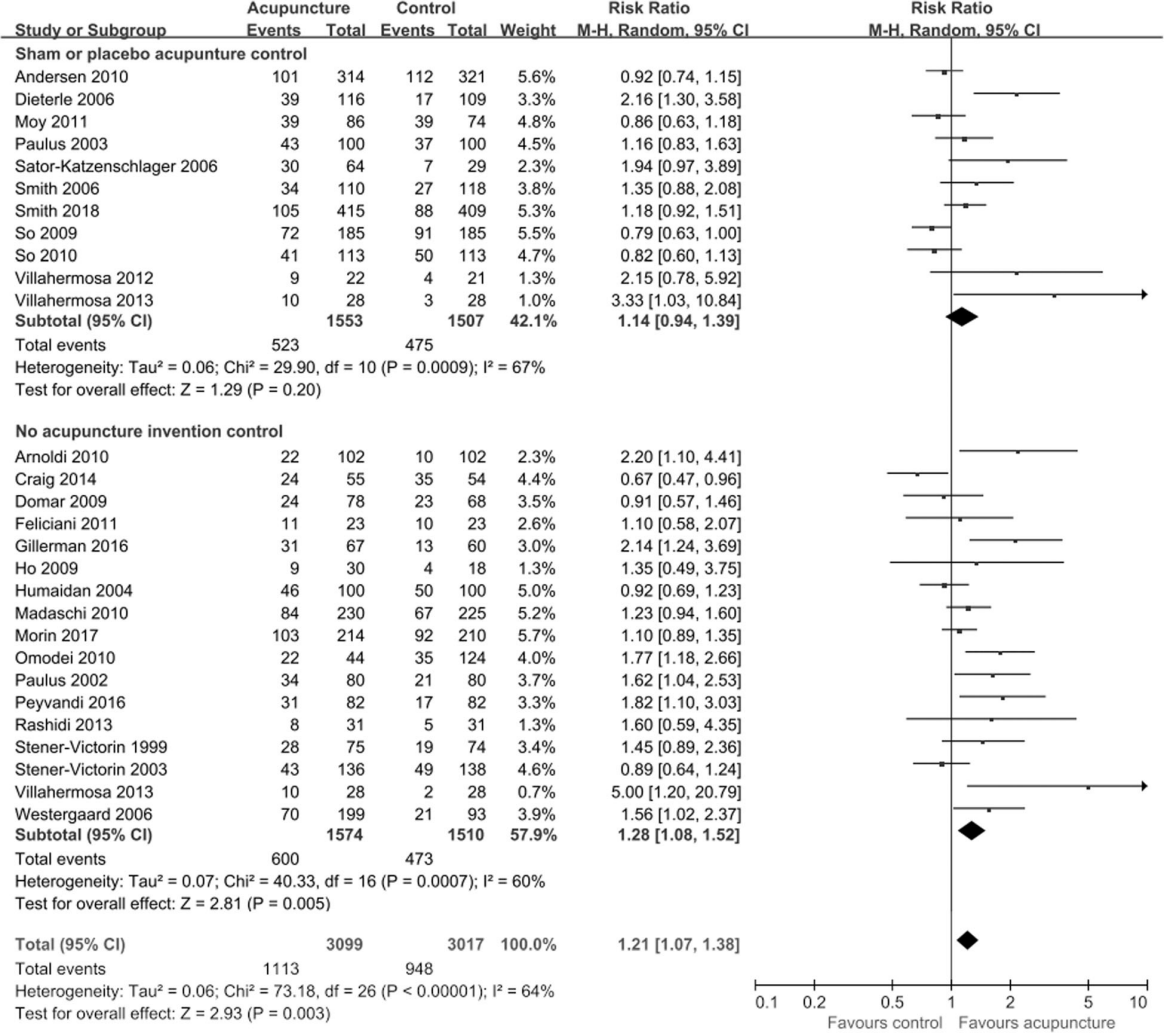
Effect of acupuncture on the clinical pregnancy rates in women undergoing IVF. Villahermosa 2013 had one acupuncture group and two control groups (sham acupuncture group and no intervention control group), two control groups were grouped separately for relevant subgroup analysis, and when calculated the total pooled risk ratio (RR), we took them together

CPR data (Fig. 2) were available from 27 trials (*n =* 6116). Pooled results showed a significant difference between acupuncture and control groups (RR = 1.21, 95% CI: 1.07–1.38, *p =* 0.003, *I*^*2*^ *=* 64%).

**Fig. 3:**
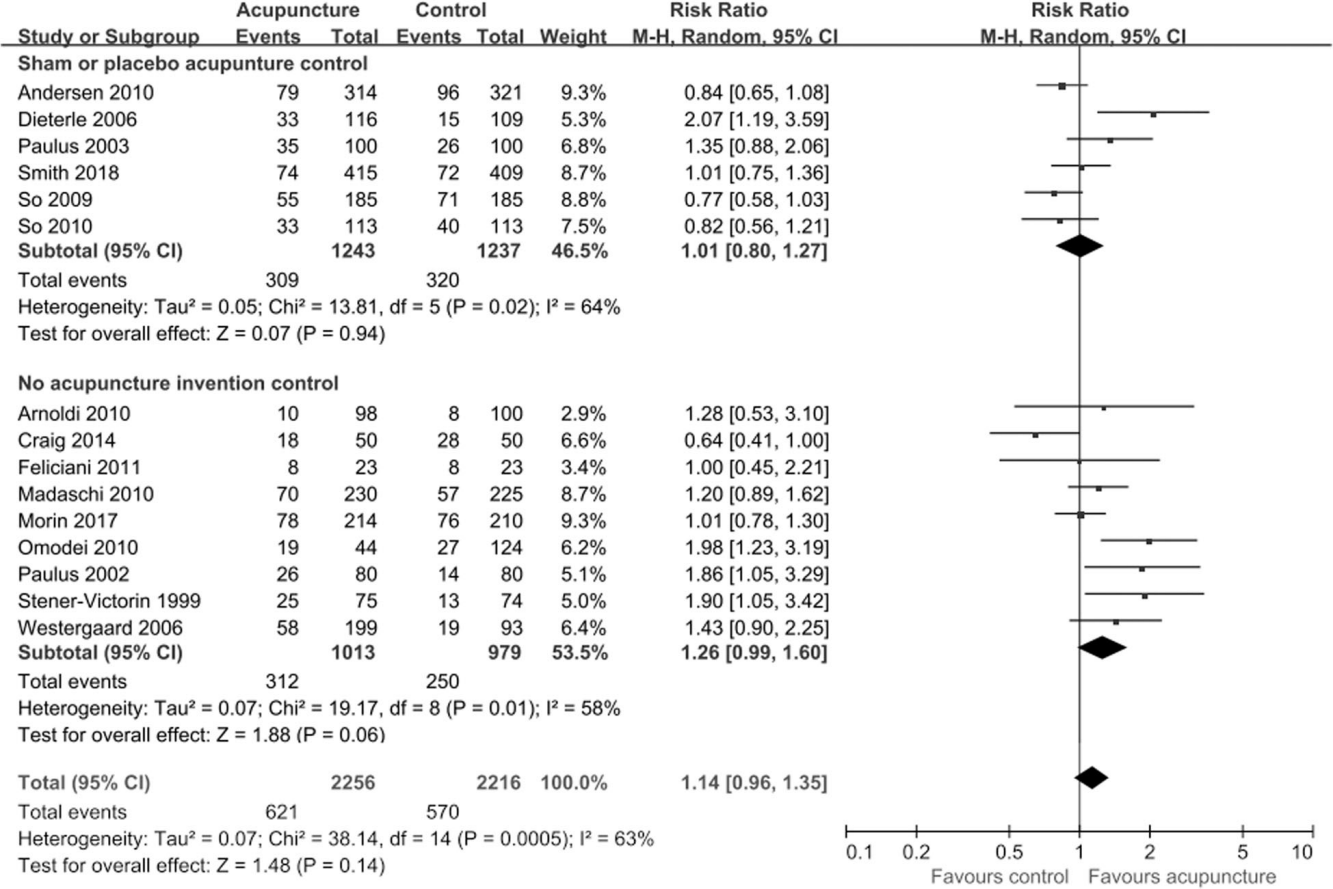
Effect of acupuncture on the live birth rates in women undergoing IVF

LBR data (Fig. 3) were available from15 trials (*n* = 4472). There was no statistically significant difference between acupuncture and control groups (RR = 1.14, 95% CI: 0.96–1.35, *p =* 0.14, *I*^*2*^ *=* 63%).

### Subgroup analysis

There was statistical heterogeneity for pregnancy outcomes across studies (CPR: χ^2^*P*-value < 0.00001, *I*^*2*^ *=* 64% and LBR: χ^2^*P*-value = 0.0005, *I*^*2*^ *=* 63%, respectively). Random-effects meta-regression and subgroup analyses were used to explore the potential sources of heterogeneity. The information on the variables for meta-regression analyses are displayed in Table 2. Meta-regression subgroup analyses were performed based on age, duration of infertility, primary infertility proportion, repeated IVF cycle proportion, number of embryos transferred, type of acupuncture invention, number of acupuncture treatments and type of control group to determine the possible effects of these variables.

**Table 2:** The information of variables for meta-regression subgroup analyses

Study	Age (years)	Duration of infertility (years)	Repeated IVF cycles(%)	No. of embryos transferred	Primary infertility(%)	No. of acupuncture sessions	Acupuncture type	Control
Andersen 2010 [65]	31.0	2.5	42.4%	1.39	71%	1	MA	SC
Arnoldi 2010 [18]			100%	2.10		2	MA	NC
Craig 2014 [59]	33.1			2.10		1	MA	NC
Dieterle 2006 [15]	34.9	5.4	83.6%^a^	2.60	80%	2	MA	SC
Domar 2009 [29]	36.1			2.51		1	MA	NC
Feliciani 2011 [62]	34.9			1.84		3	MA	NC
Gillerman 2016 [58]						3	MA	NC
Ho 2009 [17]	35.0			3.56		4	EA	NC
Humaidan 2004 [69]	31.0					1	EA	NC
Madaschi 2010 [64]	35.0			2.15		1	MA	NC
Morin 2017 [59]	34.5			2.57		1	MA	NC
Moy 2011 [61]	33.2			2.15	63%	1	MA	SC
Omodei 2010 [19]	35.7			2.05		1	MA	NC
Paulus 2002 [14]	32.5			2.15		1	MA	NC
Paulus 2003 [71]	32.6					1	MA	SC
Peyvandi 2016 [22]						3	EA	NC
Rashidi 2013 [60]	31.6	9.3		2.55	76%	4	EA	NC
Sator-K 2006 [68]	33.8		77.7%			1	EA/MA	SC
Smith 2006 [67]	36.0	2.9	52.2%		76%	2	MA	SC
Smith 2018 [56]	35.4		70%	1.32	74%	2	MA	SC
So 2009 [66]	36.0	4.0	30.5%	1.89	61%	1	MA	SC
So 2010 [63]	36.0	5.0		1.93	38%	1	MA	SC
Stener-Victorin 1999 [72]	33.4		60.4%	2.03		1	EA	NC
Stener-Victorin 2003 [70]	32.9		47.8%	1.92		1	EA	NC
Villahermosa 2012 [20]	34.2	4.0				4	MA	SC
Villahermosa 2013 [21]	36.2	4.5	100%	2.13	82%	4	MA	SC/OC^b^
Westergaard 2006 [16]	37.0	3.7	73.6%	2.07	42%	1/2^c^	MA	NC

Note: *MA* manual acupuncture, *EA* electro acupuncture, *SC* sham control, *NC* no intervention control

^a^Dieterle 2006 reported clinical pregnancy rates for first, second and more than two IVF cycles, and then we grouped these three arms separately for meta-regression subgroup analyses

^b^This trial had two control groups, sham acupuncture group and no intervention control group,and these groups were grouped separately for relevant subgroup analysis

^c^Westergarrd 2006 had two acupuncture treatment arms, one arm received two sessions and the other arm received three, and these arms were grouped separately for this meta-regression

**Fig. 4:**
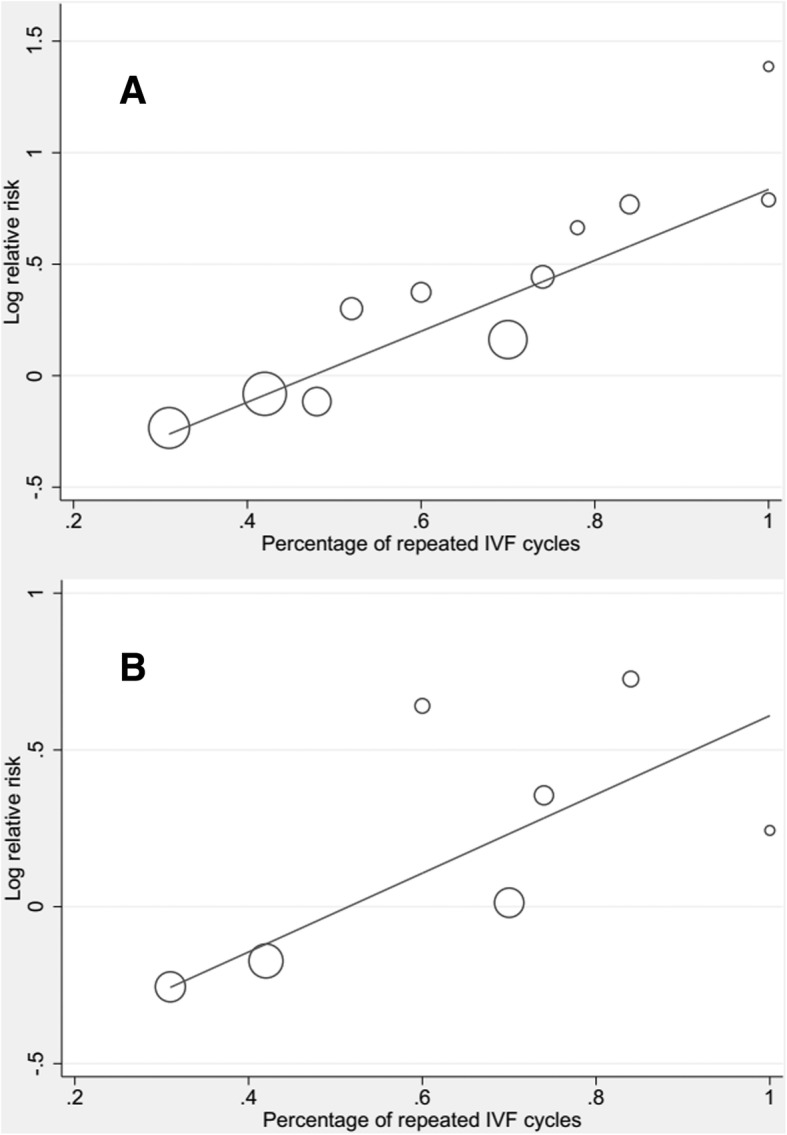
Meta-regression between the effects of acupuncture on clinical pregnancy rates (**a**)/ live birth rates (**b**) and repeated IVF cycle proportion as a single continuous covariate

Repeated IVF cycle proportion (number of women with a history of unsuccessful IVF attempt before included divided by number of women included in each trial) was reported in 11 studies [15, 18, 21, 56, 63, 65–68, 70, 72]. In subgroups classified by repeated IVF cycle proportion (≥ 50% or < 50%), a significant outcome was found in the repeated IVF cycle proportion ≥ 50% group (CPR: RR = 1.60, 95% CI: 1.28–2.00; LBR: RR = 1.42, 95% CI: 1.05–1.92, Table 3). The results of univariate meta-regressions indicated that pooled RR significantly varied with repeated IVF cycle proportion, and a positive correlation between the two was detected (Fig. 4). It was also the major sources of heterogeneity explained the great mass of the between-study variance for pregnancy outcomes (CPR: *p <* 0.001, adjusted *R*^*2*^ *=* 100%, *I*^*2*^ residual *=* 0%; LBR: *p =* 0.046, adjusted *R*^*2*^ *=* 87.90%, *I*^*2*^ residual *=* 32.78%; Table 3).

**Table 3:** The results of meta-regression subgroup analyses for primary outcomes

Characteristic	Subgroup analyses					Meta-regression			
	No. of subjects	No. of studies	Random-effects RR (95% CI)	Heterogeneity		Coefficient	*p*-value	I^2^_resid_	Adj R^2^
				I^2^	*P*				
**Clinical pregnancy**									
Age									
< 33.3 years	1800	8	0.96 (0.82, 1.13)	42.1%	0.097	0.045	0.270	60.38%	0.71%
≥ 33.3 years	3821	16	1.27 (1.08, 1.50)	62%	< 0.005				
Duration of infertility									
< 5. 6 years	2103	8	1.27 (0.95, 1.71)	77.6%	< 0.001	0.072	0.523	76.70%	−5.06%
≥ 5. 6 years	62	1	1.60 (0.59, 4.35)	/	/				
Percentage of primary infertility									
< 50%	518	2	1.11 (0.59, 2.10)	82.7%	0.016	1.252	0.203	71.19%	2.77%
≥ 50%	2588	8	1.18 (0.92, 1.52)	73.7%	< 0.001				
Percentage of repeated IVF cycle									
< 50%	1279	3	0.86 (0.75, 1.00)	0%	0.63	1.59	< 0.001	0%	100%
≥ 50%	2099	8	1.60 (1.28, 2.00)	39%	0.12				
No. of embryos transferred									
< 1.9	1875	4	0.96 (0.79, 1.16)	46%	0.14	0.219	0.305	65.92%	0.79%
≥ 1.9	3186	16	1.24 (1.03, 1.49)	67.3%	< 0.001				
Type of acupunture invention									
Electroacupuncture	958	7	1.30 (0.96, 1.75)	56.5%	0.032	0.066	0.713	65.29%	−6.38%
Manual acupuncture	5187	21	1.21 (1.04, 1.40)	67.8%	< 0.001				
No. of acupuncture treatments									
one session	3962	16	1.06 (0.92, 1.21)	61.1%	0.001	0.248	0.002	48.40%	51.90%
≥ two sessions	2055	12	1.60 (1.32, 1.92)	24.9%	0.199				
Type of control group									
Sham or placebo acupuncture control	3060	11	1.14 (0.94, 1.39)	66.6%	0.001	−0.109	0.466	62.34%	−0.25%
No acupuncture invention control	3084	17	1.28 (1.08 1.52)	60.3%	0.001				
**Live birth**									
Age									
< 33.3 years	895	3	0.96 (0.59, 1.58)	77%	0.013	0.026	0.693	68.74%	−13.18%
≥ 33.3 years	3179	10	1.18 (0.96, 1.45)	63%	0.003				
Duration of infertility									
< 5. 6 years	1748	5	1.03 (0.76, 1.40)	71.6%	0.007	0.157	0.712	75.35%	−20.26%
≥ 5. 6 years	/	/	/	/	/				
Percentage of primary infertility									
< 50%	518	2	1.07 (0.62, 1.83)	69.7%	0.069	0.497	0.658	71.40%	−43.04%
≥ 50%	2054	4	1.00 (0.74, 1.36)	71.7%	0.014				
Percentage of repeated IVF cycle									
< 50%	1005	2	0.81 (0.67, 1.01)	0%	0.673	1.256	0.046	32.78%	87.90%
≥ 50%	1688	5	1.42 (1.05, 1.92)	45%	0.12				
No. of embryos transferred									
< 1.9	1865	4	0.87 (0.74, 1.02)	0%	0.604	0.386	0.163	62.55%	7.83%
≥ 1.9	2397	10	1. 27 (1.00, 1.62)	66.0%	0.002				
Type of acupunture invention									
Electroacupuncture	149	1	1.90 (1.05, 3.42)	/	/	0.534	0.226	61.92%	9.04%
Manual acupuncture	4323	14	1.11 (0.93, 1.31)	62.0%	0.001				
No. of acupuncture treatments									
one session	3080	11	1.13 (0.92, 1.39)	70.4%	< 0.001	0.099	0.654	64.01%	−7.71%
≥ two sessions	1485	5	1.22 (0.93, 1.60)	23.2%	0.266				
Type of control group									
Sham or placebo acupuncture control	2480	6	1.01 (0.80, 1.27)	63.8%	0.017	−0.213	0.274	60.40%	7.21%
No acupuncture invention control	1992	9	1.26 (0.99, 1.60)	58.3%	0.014

In the subgroup analysis based on the number of acupuncture treatments, the results indicated that women receiving two or more treatments had a significant improvement in clinical pregnancy (CPR: RR = 1.60, 95% CI: 1.32–1.92, Table 3). The results of univariate meta-regression with clinical pregnancy rates as the dependent variables also suggested that number of acupuncture treatments was a statistically significant variable which explained 51. 90% of the heterogeneity between included studies (*p =* 0.002, adjusted *R*^*2*^ *=* 51.90%, *I*^*2*^ residual *=* 48.40%, Table 3). Unexpectedly, the meta-regression for live birth rates did not offer sufficient evidence to convince us that the number of acupuncture treatments was a covariate that led to heterogeneity (*p =* 0.654, adjusted *R*^*2*^ *=* − 7.71%, *I*^*2*^ residual *=* 64.01%, Table 3), as per our hypothesis that there was an increasing gradient of RR with increasing acupuncture treatment number.

In addition, differences in age, duration of infertility, type of infertility, number of embryos transferred, type of acupuncture invention and control fail to explain a large proportion of the between trial variability. Detailed results of meta-regression subgroup analyses are displayed in Table 3.

### Side effects

Fifteen trials evaluated the possible impact of acupuncture on miscarriage rates. Using the random-effects model, pooling of the results from the 15 trials showed no significant difference in the miscarriage outcome between the acupuncture and control groups (RR =1.14, 95% CI: 0.93–1.41; Additional file 2: Figure S2).

### Sensitivity analyses

The analyses described above were repeated under the four scenarios: (1) when we included randomized participants whose clinical pregnancy outcome data were missing, the above finding were not affected; (2) when acupuncture protocol which performed pre- and post- ET was regarded as two acupuncture sessions, the results for subgroup analysis and meta-regression remain unchanged (CPR: *p* = 0.008; LBR: *p* = 0.817); (3) when we restricted CPR to the 15 studies which reported LBR, the pooled results returned no difference between acupuncture and control groups for CPR (Additional file 3: Figure S3). In meta-regression and subgroup analyses, the repeated IVF cycle proportion remained a statistically significant effect modifier (*p* = 0.006), but the number of acupuncture treatments did not (*p* = 0.240; Additional file 5: Table S2); (4) we also analyzed sham-controlled and no adjuvant treatment controlled trials separately. However, since there were no large or significant differences between these two subsets in LBR (Table 3), as well as the largely similar subgroup effects across the two control groups, we preferred to conduct meta-regression and subgroup analyses stratified by control type for CPR. There were no significant changes in the results: the repeated IVF cycle proportion and number of acupuncture treatments remained statistically significant effect modifiers whenever restricted to no acupuncture invention control (*p* = 0.049 and 0.035) or sham (placebo) acupuncture control (*p* = 0.009 and 0.010; Additional file 6: Table S3).

In addition, we performed the meta-regression subgroup analyses according to ‘risk of bias’ of included studies. When we restricted study eligibility to “low risk of bias”, the results remained stable (Additional file 7: Table S4).

### Publication bias

**Fig. 5:**
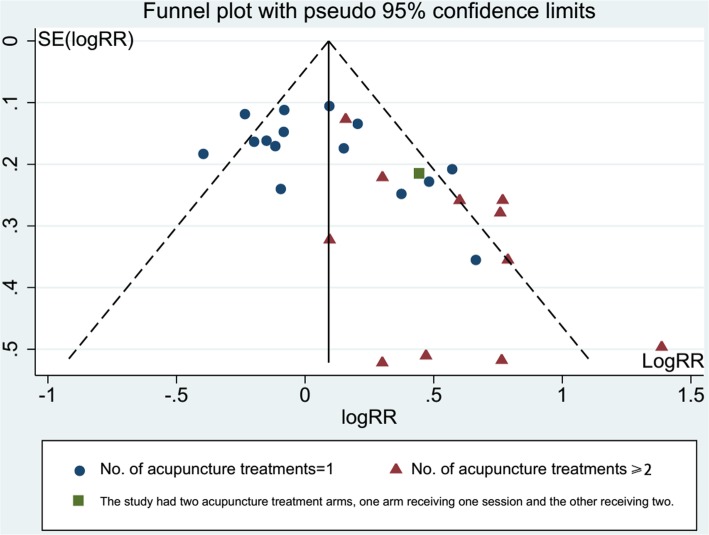
The funnel plot for the 27 eligible studies (Begg’s and Egger’s test, *p* = 0.006). The studies are separated into two groups by No. of acupuncture treatments either one or more than one

Funnel plot analysis showed that there were no significant publication biases for most analyses, except for acupuncture compared with all controls for clinical pregnancy rates (Fig. 5). Both Begg’s and Egger’s test results supported the possible of publication bias (*p* = 0.006).

### Levels of evidence

Overall, the levels of evidence as determined by GRADE were very low for the pooled CPR (27 studies) and LBR (15 studies) from all the acupuncture groups compare with control groups. For miscarriage rates, moderate quality of evidence was found. In stratified analyses, moderate quality of evidence showed that acupuncture improved IVF outcomes among women with a history of unsuccessful IVF attempt. However, the levels of evidence for other subgroup analyses were found to be from very low to low.

## Discussion

### Main findings

The objective of this review was to summarize and evaluate the effects of acupuncture on pregnancy outcomes among women undergoing IVF. Compared with controls, we found a low level of evidence that acupuncture significantly increased CPR among women undergoing IVF (RR = 1.21, 95% CI: 1.07–1.38), but not LBR. However, there was substantial heterogeneity between these trials. Subsequently, in subgroup analyses classified by the repeated IVF cycle proportion (≥ 50% or < 50%) and number of acupuncture treatments, we found a moderate level of evidence that acupuncture improved IVF outcomes in repeated IVF cycle proportion ≥ 50% subset (CPR: RR = 1.60, 95% CI: 1.28–2.00; LBR: RR = 1.42, 95% CI: 1.05–1.92), as well as in more than one acupuncture treatment subset for CPR (RR = 1.60, 95% CI: 1.32–1.92). And the univariate meta-regression suggested a positive correlation between the pooled RR and repeated IVF cycle proportion (CPR: *p <* 0.001; LBR: *p =* 0.046), it was also the major sources of heterogeneity (CPR: adjusted *R*^*2*^ *=* 100%; LBR: adjusted *R*^*2*^ *=* 87.90%). Similar results were found between the pooled CPR and number of acupuncture treatments in the meta-regression model (*p =* 0.002, adjusted *R*^*2*^ *=* 51.90%, *I*^*2*^ residual *=* 48.40%).

Moreover, the results of sensitivity analysis were also consistent with the findings described above, except for the scenario when CPR results were restricted to the 15 studies which reported LBR. In that case the results of the meta-regression between the pooled CPR and number of acupuncture treatments turned negative.

### Interpretation

This review indicates that acupuncture improves CPR rather than LBR. Firstly, we considered this suggested a higher risk of miscarriage. Fifteen trials evaluated the possible impact of acupuncture on miscarriage rates, however, we did not find statistical evidence for this (RR =1.14, 95% CI: 0.93–1.41; Additional file 2: Figure S2). Secondly, it may be caused by the missing information of LBR. Only 15 among 27 studies reported both CPR and LBR, when we restricted CPR to the 15 studies which reported LBR, the pooled results returned no difference between acupuncture and control groups for CPR (Additional file 3: Figure S3). One plausible reason for this variation could be the heterogeneity (population-, treatment- and study design-related varieties) among trials. In our subgroup analyses and sensitivity analyses, we found a significantly increased CPR and LBR in two or more acupuncture treatments and the repeated IVF cycle proportion ≥ 50% subsets, but not in one acupuncture treatment and < 50% subsets (Table3), and when we restricted CPR results to the 15 studies which reported LBR, the results remained stable (Additional file 5: Table S2). Therefore, heterogeneity among trials may be one of possible explanations for the difference of statistical significance between CPR and LBR.

### Meta-regression subgroup analyses based on clinical characteristics

We found that patients with repeated IVF cycles benefit more from acupuncture than do women with first IVF cycle acupuncture. Repeated IVF cycle usually implies at least one implantation failure, which may be a consequence of embryo or endometrial factors [74–77], as well as the increased vulnerability to stress [78–80]. From a Chinese medical perspective, the primary principles of fertility treatment with acupuncture are to harmonize the function of the internal organs, clear obstructions and improve the qi and blood supply within the abdominal environment in order to improve egg quality and the receptiveness of the endometrium [81]. On one hand, numerous studies have shown that acupuncture can stimulate ovulation by adjusting endocrine function of the hypothalamic–pituitary–ovarian axis in women with an ovulation disorder [24, 82–84], as well as improve egg quality [60, 85], which may related to a beneficial regulation of TNF-α levels [85]. On the other hand, acupuncture improves blood circulation to the uterus and ovaries by inhibiting uterine central sympathetic nerve activity, and thereby optimizing endometrial receptivity [17, 27, 86, 87]. Moreover, acupuncture may be a useful intervention to reduce infertility-related stress and depression by changing the stress hormones (serum cortisol and prolactin) [28–30, 88–91]. Thus, as a corollary, women undergoing repeated IVF cycles may benefit more from acupuncture, which is consistent with our meta-analysis. However, the exact mechanism of this effect is still poorly understood. As repeated IVF cycle is a proxy for implantation failure, which associated with age, duration and cause of infertility, number of embryos transferred, embryo quality [48, 92–96], future studies should further investigate the relationship between these parameters and the efficacy of acupuncture on IVF outcomes.

Although meta-regression subgroup analyses provided insufficient evidence to support hypotheses regarding the age, duration of infertility and number of embryos transferred, this may due to aggregation bias. These data were extracted in the form of mean values, which can only be summarized at the level of the individual study. This makes the effect of a characteristic not always identifiable due to the ecological fallacy. For example, there may be a strong relationship between age and treatment effect that is apparent within each study, however, if the mean ages for the trials are similar, then no relationship will be apparent by looking at trial mean ages and trial-level effect estimates [47]. Analyses of pooled, patient-level data will be useful to find the true role of these parameters on the efficacy of acupuncture in future studies.

### Meta-regression subgroup analyses based on methodological characteristics

The quality of included studies is an influential factor in our meta-analysis. In our review, we found a statistically significant subgroup effect for the type of control on CPR, suggesting some placebo effect. As our outcomes are entirely objective, and are unlikely to be affected by placebo effects [97–100], we also consider explanations such as heterogeneity among trials. Therefore, we conducted subgroup analyses stratified by the number of acupuncture treatments in addition to control type for CPR. However, the pooled results showed no difference between sham (placebo) acupuncture control and no acupuncture invention control when restricted to studies which performed one acupuncture treatment (95% CI: 0.79–1.09 and 0.95–1.36), or more than one acupuncture treatment (95% CI: 1.13–2.17 and 1.36–2.15). This suggests a minor contribution of placebo effect for IVF outcomes.

Recent studies have shown that sham and placebo acupuncture are sometimes therapeutically effective [101–103]. Sham or placebo acupuncture usually consists of non-insertion or superficial insertion in related acupoints or needle insertion at unrelated acupoints or non-acupoints. However, all evoke activity in cutaneous afferent nerves [101, 102]. It has been reported that placebos have small benefits in studies where continuous subjective outcomes are measured (especially patient-reported outcomes), but there are no significant effects on objective or binary outcomes [97–100]. More accurately, for objectively measured outcome parameters, placebo effects can improve physical processes (e.g., blood flow to the uterus) more easily and effectively than biochemical processes (e.g., endogenous hormone release) [104]. Consequently, it is more plausible to cautiously use sham acupuncture for subjective, patient-reported outcomes and objectively measured physical parameters before its use or non-use is established.

### Limitations

**Fig. 6:**
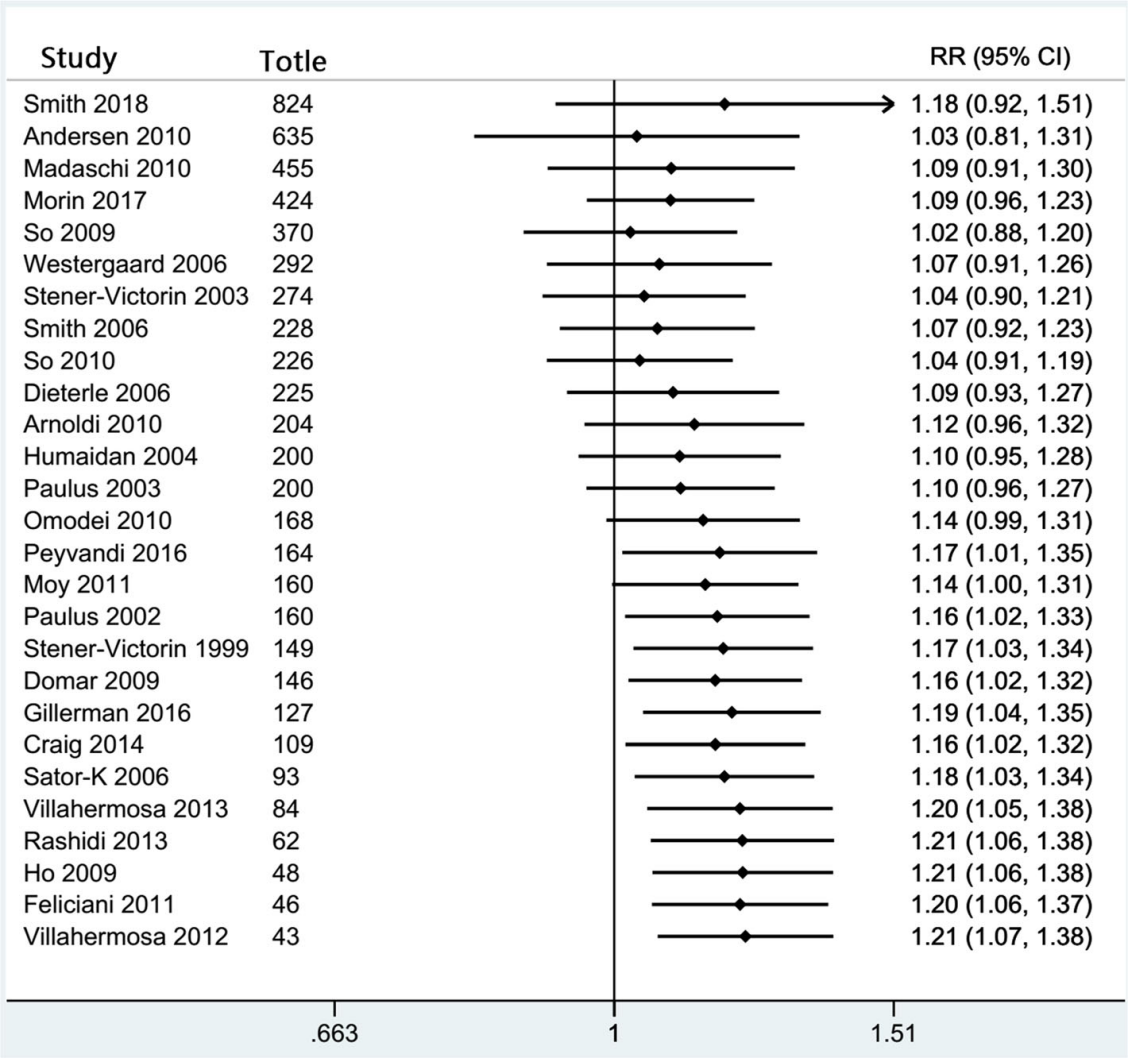
The result of cumulative meta-analysis based on sample size

Our study has a number of limitations. Firstly, the funnel plot of the effects of acupuncture on clinical pregnancy rates (Fig. 5) indicates some small-study effects – a tendency for the intervention effects estimated in smaller studies to differ from those estimated in larger studies [105]. The Begg’s and Egger’s test results (*p =* 0.006) support the finding of small study-effects that overestimate the treatment effect. However, the purpose of the test was not to test for publication bias alone, the reasons other than publication bias should also be considered, including other language bias, citation bias, time lag bias and multiple publication bias [106, 107]. We imposed no restrictions on publication type or language of publication, scanned the digital databases, the conference proceedings and reference lists of relevant primary and review articles. In addition, we corresponded with study investigators to clarify further data, so it seems that the review is less likely to be subject to the other reported biases. According to the Cochrane guidelines, “true heterogeneity in treatment effect may also lead to funnel plot asymmetry” [107]. For example, substantial benefit may be seen only in patients at high risk for the outcome which is affected by the intervention [108, 109]. Furthermore, some interventions may have been implemented less thoroughly in larger trials, therefore, have resulted in smaller estimates of the intervention effect [110]. In our analysis, we found that women with a history of unsuccessful IVF attempt, as well as having received more than one acupuncture treatment, will benefit more from the effects of acupuncture on clinical pregnancy rates. Given that only 11 studies provided the date of repeated IVF cycle proportion, we therefore added the number of acupuncture treatments to the funnel plot, and the resulting plot shows that the trials with more than one acupuncture treatment indicate more positive results (Fig. 5). We also checked separate funnel plots and conducted corresponding separate Egger tests for one and more than one acupuncture treatment trial subgroups. Ultimately, no evidence of publication bias was found when restricting to one or more than one acupuncture treatment trial subset (Begg’s and Egger’s tests for one acupuncture treatment: *p* = 0.137 and 0.07; for more than one acupuncture treatment: *p* = 0.451 and 0.06, respectively). Therefore, less thoroughly implemented or high risk patients for pregnancy outcomes affected by acupuncture may be one of many possible explanations for the funnel plot asymmetry. That is, asymmetry may be partly due to the existence of true heterogeneity. A cumulative meta-analysis was conducted to clarify the contribution of small-study effects on the results based on the sample size (Fig. 6). The cumulative effect size increased gradually after the first study with the biggest sample size (824), but reached a significant steady state at sample size 164. However, analysis by excluding the studies with small sample sizes (< 100) still showed a statistically significant difference (RR = 1.16, 95% CI: 1.02–1.32). These results indicate a minor contribution of publication bias on effect sizes.

Secondly, there were 27 studies reporting clinical pregnancy outcomes, but only 15 among 27 studies reported live birth rates. Therefore, the deficiency of IVF outcomes may lead to substantial variation, which resulted in imprecise RR and unclear associations between the strength of RR and related variables.

Lastly, the differences in acupuncture protocols were unidentified confounders which complicated the interpretation of the efficacy of acupuncture on IVF outcomes. For acupuncture time, several systematic reviews reported that IVF outcomes improved significantly when acupuncture was conducted during COH, but not ET and OA [38, 41, 42]. Moreover, from a Chinese medicine perspective, acupoints should be chosen based on the different signs and symptoms of individuals. This may account for the results of Cui et al.’s study, where women with three different syndromes were treated with the same acupuncture protocol. They showed that the effects of acupuncture on IVF-ET in women with kidney deficiency type and liver-qi stagnation type were better than those of phlegm-dampness type [111]. In addition, the frequency, duration and the mode of stimulation of acupuncture treatment may also influence to the efficacy of acupuncture. Fortunately, a Delphi consensus protocol was published to establish the parameters of best acupuncture practice for ART in 2012. In future studies this protocol may be helpful to clarify the efficacy of acupuncture on IVF outcomes.

### Comparison with other studies and reviews

We acknowledge that there are several systematic reviews and meta-analyses analyzed the effects of acupuncture among women undergoing IVF [31–43]. However, they showed differences in their results and conclusions. The reasons for these discrepancies may arise from the heterogeneity across studies, such as variation in participants, interventions, trial design and quality. And few studies try to investigate sources of heterogeneity. In our meta-analysis, we attempted pre-specified meta-regression subgroup analyses to explore the influences of eight variables on the effects of acupuncture on both clinical pregnancy and live birth outcomes. Ultimately, we revealed some new findings. First of all, there is a significant benefit of adjuvant acupuncture on both CPR and LBR for women with a history of unsuccessful IVF attempt. Secondly, number of acupuncture treatments is a promising influential factor for the effect estimates. In addition, our search identified four additional studies [22, 56–58], which were not included in the earlier reviews.

## Conclusion

Our analysis finds a benefit of acupuncture for IVF outcomes among women with a history of unsuccessful IVF attempt, and number of acupuncture treatments is a potential influential factor. As repeated IVF cycle is possibly a proxy for implantation failure, which associated with poor prognoses (i.e. increasing age, longer history of infertility, elevated Peak FSH). In the future, analyses of pooled, patient-level data will be useful to find the true role of these parameters on the efficacy of acupuncture on IVF outcomes.

## Additional files

Additional file 1: **Figure S1.** Study selection PRISMA flow diagram. (TIF 5172 kb)

Additional file 2: **Figure S2.** Effects of acupuncture on spontaneous abortion outcome. (TIF 1233 kb)

Additional file 3: **Figure S3.** Effects of acupuncture on clinical pregnancy rates for 15 studies which reported live birth rates. (TIF 1519 kb)

Additional file 4: **Table S1.** Search strategy for the MEDLINE Database. (DOC 50 kb)

Additional file 5: **Table S2.** The results of meta-regression subgroup analyses for the 15 studies which reported LBR. (DOC 61 kb)

Additional file 6: **Table S3.** The results of meta-regression subgroup analyses stratified by control type. (DOC 84 kb)

Additional file 7: **Table S4.** The results of meta-regression subgroup analyses according to ‘risk of bias’ for primary outcomes. (DOC 73 kb)

Additional file 8: **Appendix 1.** The reasons for exclusion studies. (DOC 175 kb)

## Abbreviations

CI: Confidence intervals
COH: Controlled ovarian hyperstimulation
CPR: Clinical pregnancy rate
DR: Delivery rates
EA: Electrical acupuncture
ET: Embryo transfer
ICSI: Intracytoplasmic sperm injection
IVF: In vitro fertilization
LBR: Live birth rate
MA: Manual acupuncture
PCOS: Polycystic ovary syndrome
RCTs: Randomized controlled trials
RR: Relative risks
TCM: Traditional Chinese medicine
