# Supplementary figures and images for: The effects of acupuncture on pregnancy outcomes of in vitro fertilization: a systematic review and meta-analysis
Source: BMC Complement Altern Med. 2019 Jun 14;19:131. doi: 10.1186/s12906-019-2523-7 (PMC6570865; doi:10.1186/s12906-019-2523-7)

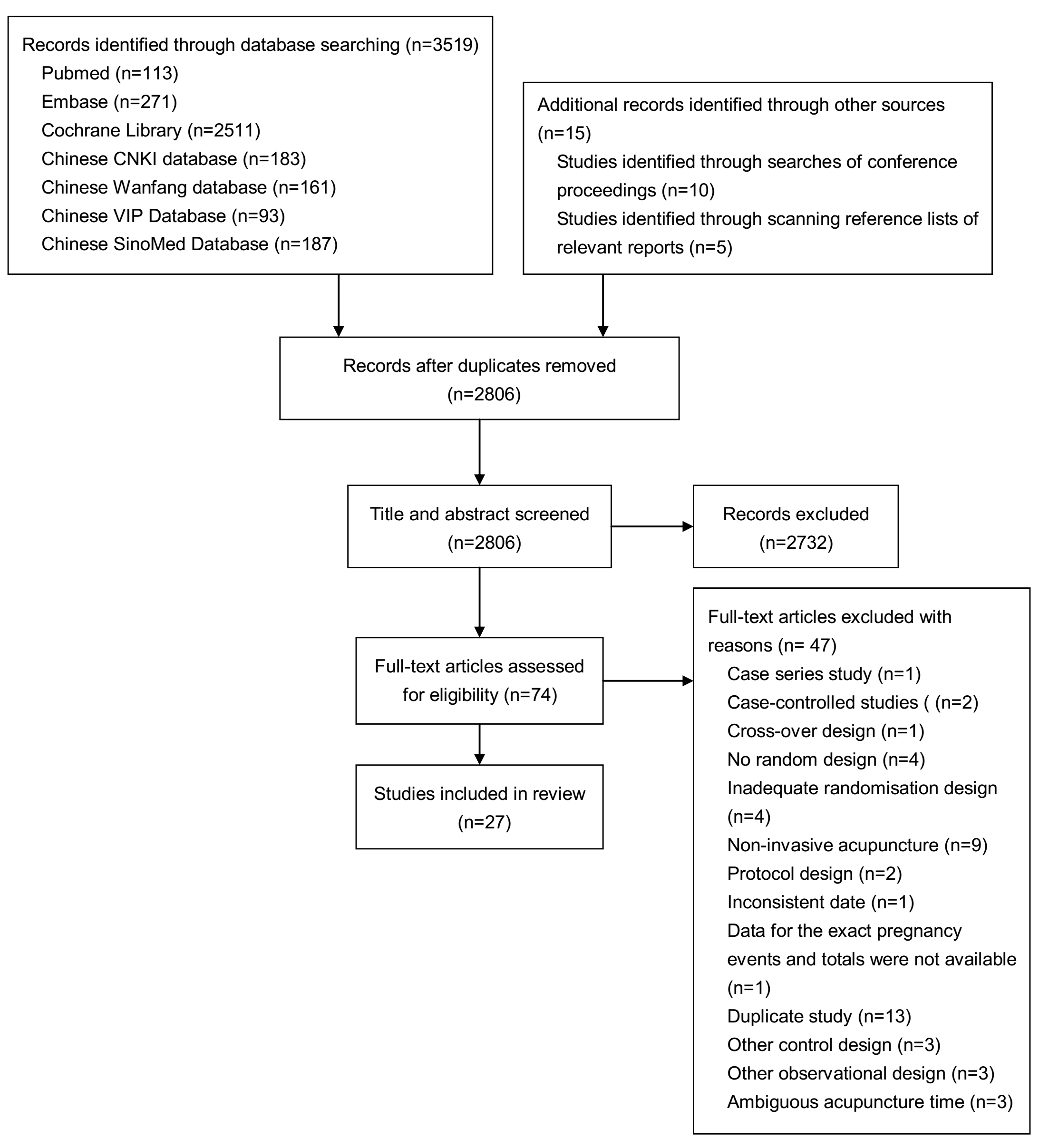

Supplement: Supplementary file 1 — Figure S1. Study selection PRISMA flow diagram. (TIF 5172 kb) [file 12906_2019_2523_MOESM1_ESM.tif]

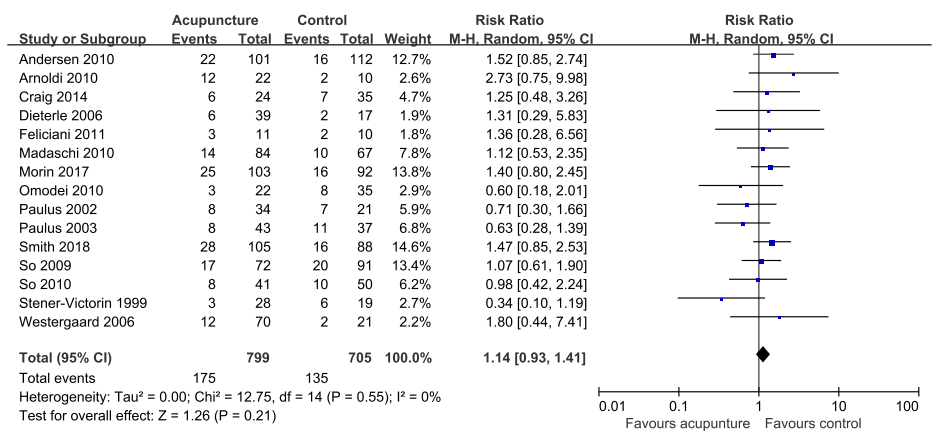

Supplement: Supplementary file 2 — Figure S2. Effects of acupuncture on spontaneous abortion outcome. (TIF 1233 kb) [file 12906_2019_2523_MOESM2_ESM.tif]

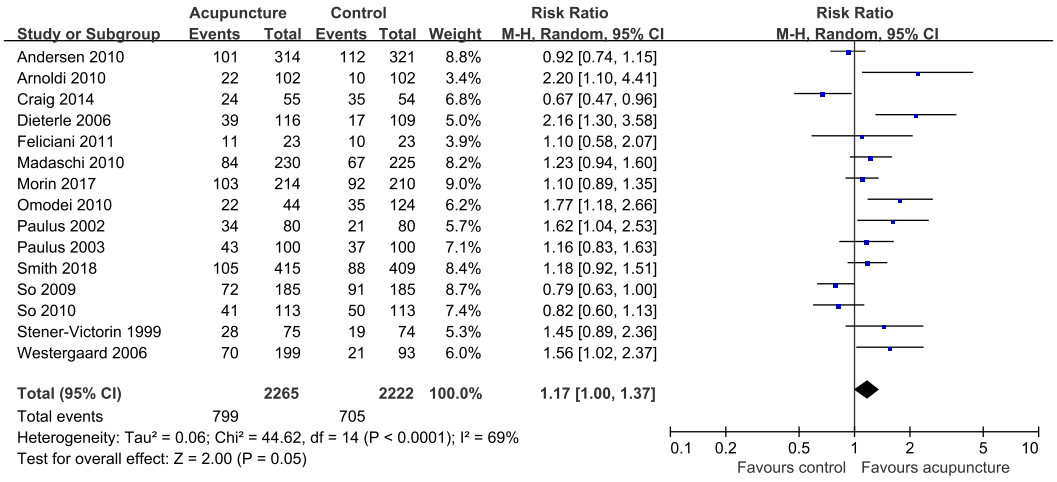

Supplement: Supplementary file 3 — Figure S3. Effects of acupuncture on clinical pregnancy rates for 15 studies which reported live birth rates. (TIF 1519 kb) [file 12906_2019_2523_MOESM3_ESM.tif]
