# Supplementary material for: The effects of acupuncture on pregnancy outcomes of in vitro fertilization: a systematic review and meta-analysis
Source: BMC Complement Altern Med. 2019 Jun 14;19:131. doi: 10.1186/s12906-019-2523-7 (PMC6570865; doi:10.1186/s12906-019-2523-7)
Supplement: Supplementary file 4 — Table S1. Search strategy for the MEDLINE Database. (DOC 50 kb) [file 12906_2019_2523_MOESM4_ESM.doc]

| **Additional file 4: Table S1. Search strategy for the MEDLINE Database.** | | |  | |
| --- | --- | --- | --- | --- |
| Pubmed (1966 - December 2018) | | |  | |
| Search | Query | Items found | |  |
| #1 | Search ("Acupuncture"[Mesh]) OR "Acupuncture Therapy"[Mesh] | 23013 | |  |
| #2 | Search Acupuncture | 29574 | |  |
| #3 | Search Electroacupuncture | 4879 | |  |
| #4 | Search Auriculotherapy | 498 | |  |
| #5 | Search("Auriculotherapy"[Mesh]) OR "Acupuncture,Ear"[Mesh] | 429 | |  |
| #6 | Search auricular acupuncture | 1260 | |  |
| #7 | Search moxibustion | 4898 | |  |
| #8 | Search Acupuncture Analgesia | 2442 | |  |
| #9 | Search acupoint | 9150 | |  |
| #10 | Search Acup* | 29645 | |  |
| #11 | Search ((((((((((("Acupuncture"[Mesh]) OR "Acupuncture Therapy"[Mesh])) OR Acupuncture) OR Electroacupuncture) OR Auriculotherapy) OR "Auriculotherapy"[Mesh]) OR auricular acupuncture) OR moxibustion) OR Acupuncture Analgesia) OR acupoint) OR Acup* | 31300 | |  |
| #12 | Search "Reproductive Techniques, Assisted"[Mesh] | 65485 | |  |
| #13 | Search Embryo Transfer | 25992 | |  |
| #14 | Search Assisted Reproducti* | 15364 | |  |
| #15 | Search Assisted Reproductive Technology | 69171 | |  |
| #16 | Search in Vitro Fertili* | 23081 | |  |
| #17 | Search intracytoplasmic sperm injection | 9552 | |  |
| #18 | Search Embryo Implantation | 18746 | |  |
| #19 | Search egg collection | 1833 | |  |
| #20 | Search Oocyte Retrieval | 3995 | |  |
| #21 | Search oocyte | 77601 | |  |
| #22 | Search oocytes | 65917 | |  |
| #23 | Search ((((((((((oocytes) OR oocyte) OR Oocyte Retrieval) OR egg collection) OR Embryo Implantation) OR intracytoplasmic sperm injection) OR in Vitro Fertili*) OR Assisted Reproductive Technology) OR Assisted Reproducti*) OR Embryo Transfer) OR "Reproductive Techniques, Assisted"[Mesh] | 149363 | |  |
| #24 | Search ((((((((((((oocytes) OR oocyte) OR Oocyte Retrieval) OR egg collection) OR Embryo Implantation) OR intracytoplasmic sperm injection) OR in Vitro Fertili*) OR Assisted Reproductive Technology) OR Assisted Reproducti*) OR Embryo Transfer) OR "Reproductive Techniques, Assisted"[Mesh])) AND (((((((((((("Acupuncture"[Mesh]) OR "Acupuncture Therapy"[Mesh])) OR Acupuncture) OR Electroacupuncture) OR Auriculotherapy) OR "Auriculotherapy"[Mesh]) OR auricular acupuncture) OR moxibustion) OR Acupuncture Analgesia) OR acupoint) OR Acup*) | 232 | |  |
| #25 | Search ((randomized controlled trial [pt] OR controlled clinical trial [pt] OR randomized [tiab] OR placebo [tiab] OR clinical trials as topic [mesh: noexp] OR randomly [tiab] OR trial [ti]) NOT (animals [mh] NOT humans [mh])) | 1112394 | |  |
| #26 | Search ((((randomized controlled trial [pt] OR controlled clinical trial [pt] OR randomized [tiab] OR placebo [tiab] OR clinical trials as topic [mesh: noexp] OR randomly [tiab] OR trial [ti]) NOT (animals [mh] NOT humans [mh])))) AND (((((((((((((oocytes) OR oocyte) OR Oocyte Retrieval) OR egg collection) OR Embryo Implantation) OR intracytoplasmic sperm injection) OR in Vitro Fertili*) OR Assisted Reproductive Technology) OR Assisted Reproducti*) OR Embryo Transfer) OR "Reproductive Techniques, Assisted"[Mesh])) AND (((((((((((("Acupuncture"[Mesh]) OR "Acupuncture Therapy"[Mesh])) OR Acupuncture) OR Electroacupuncture) OR Auriculotherapy) OR "Auriculotherapy"[Mesh]) OR auricular acupuncture) OR moxibustion) OR Acupuncture Analgesia) OR acupoint) OR Acup*)) | 113 | |  |
