# Supplementary material for: The effects of acupuncture on pregnancy outcomes of in vitro fertilization: a systematic review and meta-analysis
Source: BMC Complement Altern Med. 2019 Jun 14;19:131. doi: 10.1186/s12906-019-2523-7 (PMC6570865; doi:10.1186/s12906-019-2523-7)
Supplement: Supplementary file 5 — Table S2. The results of meta-regression subgroup analyses for the 15 studies which reported LBR. (DOC 61 kb) [file 12906_2019_2523_MOESM5_ESM.doc]

| **Table S2** **The results of meta-regression subgroup analyses** **for the 14 studies** **which reported LBR.** | | | | | | | | | |
| --- | --- | --- | --- | --- | --- | --- | --- | --- | --- |
| **Clinical pregnancy** |  |  |  |  |  |  |  |  |  |
| **Characteristic** | **Subgroup analyses** | | | | | **Meta-regression** | | | |
|  | **No. of subjects** | **No. of studies** | **Random-effects RR (95 % CI)** | **Heterogeneity** | | **Coefficient** | ***p*-value** | **I^2^_resid_** | **Adj R^2^** |
|  |  |  |  | **I^2^** | ***P*** |  |  |  |  |
| **Age** | | | | | | | | | |
| ＜ 33.3 years | 1104 | 4 | 1.02 (0.75, 1.38) | 71.0% | 0.016 | 0.028 | 0.612 | 69.63% | -11.40% |
| ≥ 33.3 years | 3179 | 10 | 1.20 (1.00 1.45) | 67.6% | 0.001 |  |  |  |  |
| **Duration of infertility** | | | | | | | | | |
| ＜ 5. 6 years | 1748 | 5 | 1.09 (0.80, 1.47) | 78.9% | 0.001 | 0.133 | 0.559 | 83.25% | -28.85% |
| ≥ 5. 6 years | / | / | / | / | / |  |  |  |  |
| **Percentage of primary infertility** | | | | | | | | | |
| ＜ 50% | 518 | 2 | 1.11 (0.59, 2.10) | 82.7% | 0.016 | 0.698 | 0.545 | 79.13% | -22.32% |
| ≥ 50% | 2054 | 4 | 1.09 (0.80, 1.49) | 80.2% | 0.002 |  |  |  |  |
| **Percentage of repeated IVF cycle** | | | | | | | | | |
| ＜ 50% | 1005 | 2 | 0.86 (0.73, 1.01) | 0% | 0.347 | **1.440** | **0.006** | **0%** | **100%** |
| ≥ 50% | 1694 | 5 | 1.53 (1.19, 1.96) | 39.1% | 0.161 |  |  |  |  |
| **No. of embryos transferred** | | | | | | | | | |
| ＜ 1.9 | 1875 | 4 | 0.96 (0.79, 1.16) | 46% | 0.135 | 0.321 | 0.233 | 70.75% | -0.25.% |
| ≥ 1.9 | 2412 | 10 | 1.30(1.04, 1.62) | 71.9% | < 0.001 |  |  |  |  |
| **Type of acupunture invention** | | | | | | | | | |
| Electroacupuncture | 149 | 1 | 1.45 (0.89, 2.36) | / | / | 0.220 | 0.587 | 69.66% | -5.31% |
| Manual acupuncture | 4338 | 14 | 1.16 (0.99, 1.36) | 69.9% | < 0.001 |  |  |  |  |
| **No. of acupuncture treatments** | | | | | | | | | |
| one session | 3089 | 11 | 1.10 (0.93, 1.31) | 69.2% | < 0.001 | 0.190 | 0.240 | 65.65% | 9.23% |
| ≥ two sessions | 1491 | 5 | 1.66 (1.12, 1.94) | 42.2% | 0.140 |  |  |  |  |
| **Type of control group** | | | | | | | | | |
| Sham or placebo acupuncture control | 2480 | 6 | 1.04 (0.84, 1.30) | 71.1% | 0.004 | -0.207 | 0.253 | 65.81% | 7.52% |
| No acupuncture invention control | 2007 | 9 | 1.29 (1.04 1.59) | 62.3% | 0.007 |  |  |  |  |

The results of LBR for the 14 studies which reported LBR see Table 3.
