# Supplementary material for: The effects of acupuncture on pregnancy outcomes of in vitro fertilization: a systematic review and meta-analysis
Source: BMC Complement Altern Med. 2019 Jun 14;19:131. doi: 10.1186/s12906-019-2523-7 (PMC6570865; doi:10.1186/s12906-019-2523-7)
Supplement: Supplementary file 6 — Table S3. The results of meta-regression subgroup analyses stratified by control type. (DOC 84 kb) [file 12906_2019_2523_MOESM6_ESM.doc]

| **Table S3 The results of** **meta-regression** **subgroup analyses stratified by control type.** | | | | | | | | | |
| --- | --- | --- | --- | --- | --- | --- | --- | --- | --- |
| **Clinical pregnancy (**No acupuncture invention control**)** | | | | | | | | | |
| **Characteristic** | **Subgroup analyses** | | | | | **Meta-regression** | | | |
|  | **No. of subjects** | **No. of studies** | **Random-effects RR (95 % CI)** | **Heterogeneity** | | **Coefficient** | ***p*-value** | **I^2^_resid_** | **Adj R^2^** |
|  |  |  |  | **I^2^** | ***P*** |  |  |  |  |
| **Age** | | | | | | | | | |
| ＜ 33.3 years | 805 | 5 | 0.99 (0.73, 1.32) | 61.4% | 0.035 | 0.074 | 0.164 | 46.72% | 27.93% |
| ≥ 33.3 years | 1784 | 9 | 1.28 (1.08, 1.52) | 29.8% | 0.180 |  |  |  |  |
| **Percentage of repeated IVF cycle** | | | | | | | | | |
| ＜ 50% | 274 | 1 | 0.89 (0.64, 1.24) | / | / | **2.020** | **0.049** | **0%** | **100%** |
| ≥ 50% | 701 | 4 | 1.71 (1.26, 2.33) | 10.4% | 0.341 |  |  |  |  |
| **No. of embryos transferred** | | | | | | | | | |
| ＜ 1.9 | 46 | 1 | 1.10 (0.58, 2.07) | / | / | -0.464 | 0.888 | 59.05% | -11.86% |
| ≥ 1.9 | 2547 | 13 | 1.26 (1.03, 1.52) | 59.7% | 0.003 |  |  |  |  |
| **Type of acupunture invention** | | | | | | | | | |
| Electroacupuncture | 897 | 6 | 1.17 (0.90, 1.54) | 43.9% | 0.113 | -0.101 | 0.621 | 61.06% | -9.15% |
| Manual acupuncture | 2187 | 11 | 1.33 (1.06, 1.67) | 67.0% | 0.001 |  |  |  |  |
| **No. of acupuncture treatments** | | | | | | | | | |
| one session | 2278 | 10 | 1.14 (0.95, 1.36) | 62.3% | 0.004 | **0.196** | **0.035** | **47.72%** | **36.05%** |
| ≥ two sessions | 899 | 8 | 1.71 (1.36, 2.15) | 0% | 0.543 |  |  |  |  |
| Type of acupunture invention and control group were operationalized as dichotomous variables , others were operationalized as continuous variables.  Duration of infertility and percentage of primary infertility could not be explored as only three studies for each influential factor. | | | | | | | | | |
| **Table S3 The results of meta-regression subgroup analyses according to 'Type of control group' for primary outcomes.**  **Clinical pregnancy (**Sham or placebo acupuncture control**)** | | | | | | | | | |
| **Characteristic** | **Subgroup analyses** | | | | | **Meta-regression** | | | |
|  | **No. of subjects** | **No. of studies** | **Random-effects RR (95 % CI)** | **Heterogeneity** | | **Coefficient** | **p-value** | **I2resid** | **Adj R2** |
|  |  |  |  | **I2** | **P** |  |  |  |  |
| **Age** | | | | | | | | | |
| ＜ 33.3 years | 995 | 3 | 0.95 (0.81, 1.12) | 0% | 0.409 | 0.027 | 0.718 | 69.26% | -30.70% |
| ≥ 33.3 years | 2065 | 8 | 1.30 (0.97, 1.76) | 74.1% | < 0.001 |  |  |  |  |
| **Duration of infertility** | | | | | | | | | |
| ＜ 5. 6 years | 1783 | 7 | 1.17 (0.87, 1.58) | 73.9% | 0.001 | 0.125 | 0.552 | 77.42% | -28.35% |
| ≥ 5. 6 years | / | / | / | / | / |  |  |  |  |
| **Percentage of primary infertility** | | | | | | | | | |
| ＜ 50% | 226 | 1 | 0.82 (0.60, 1.13) | / | / | 1.753 | 0.072 | 56.50% | 54.68% |
| ≥ 50% | 2498 | 7 | 1.12 (0.88, 1.44) | 72.8% | 0.001 |  |  |  |  |
| **Percentage of repeated IVF cycles** | | | | | | | | | |
| ＜ 50% | 1005 | 2 | 0.86 (0.73, 1.01) | 0% | 0.347 | **1.520** | **0.009** | **0%** | **97.67%** |
| ≥ 50% | 1426 | 5 | 1.57 (1.15, 2.15) | 47.9% | 0.104 |  |  |  |  |
| **No. of embryos transferred** | | | | | | | | | |
| ＜ 1.9 | 1829 | 3 | 0.95 (0.76, 1.18) | 62.6% | 0.069 | 0.402 | 0.347 | 75.76% | -27.97% |
| ≥ 1.9 | 667 | 4 | 1. 28(0.75, 2.18) | 81.1% | 0.001 |  |  |  |  |
| **Type of acupunture invention** | | | | | | | | | |
| Electroacupuncture | 61 | 1 | 2.46 (1.21, 4.98) | / | / | 0.789 | 0.140 | 62.37% | 30.80% |
| Manual acupuncture | 3028 | 11 | 1.11 (0.92, 1.34) | 63.2% | 0.002 |  |  |  |  |
| **No. of acupuncture treatments** | | | | | | | | | |
| one session | 1684 | 6 | 0.93 (0.79, 1.09) | 41.2% | 0.130 | **0.369** | **0.010** | **32.80%** | **92.92%** |
| ≥ two sessions | 1376 | 5 | 1.57 (1.13, 2.17) | 46.6% | 0.112 |  |  |  |  |

Type of acupunture invention and control group were operationalized as dichotomous variables , others were operationalized as continuous variables.
