# Supplementary material for: The effects of acupuncture on pregnancy outcomes of in vitro fertilization: a systematic review and meta-analysis
Source: BMC Complement Altern Med. 2019 Jun 14;19:131. doi: 10.1186/s12906-019-2523-7 (PMC6570865; doi:10.1186/s12906-019-2523-7)
Supplement: Supplementary file 7 — Table S4. The results of meta-regression subgroup analyses according to ‘risk of bias’ for primary outcomes. (DOC 73 kb) [file 12906_2019_2523_MOESM7_ESM.doc]

| **Table S4 The results of meta-regression subgroup analyses according to 'risk of bias' for primary outcomes.** | | | | | | | | | |
| --- | --- | --- | --- | --- | --- | --- | --- | --- | --- |
| **Clinical pregnancy** |  |  |  |  |  |  |  |  |  |
| **Characteristic** | **Subgroup analyses** | | | | | **Meta-regression** | | | |
|  | **No. of subjects** | **No. of studies** | **Random-effects RR (95 % CI)** | **Heterogeneity** | | **Coefficient** | ***p*-value** | **I^2^_resid_** | **Adj R^2^** |
|  |  |  |  | **I^2^** | ***P*** |  |  |  |  |
| **Random sequence generation** | | | | | | | | | |
| Adequate | 4863 | 19 | 1.18(1.02, 1.38) | 67.6% | < 0.001 | 0.095 | 0.569 | 64.68% | -3.20% |
| Inadequate | 1253 | 8 | 1.31 (1.01, 1.69) | 56.8% | 0.023 |  |  |  |  |
| **Allocation concealment** | | | | | | | | | |
| Adequate | 4452 | 18 | 1.14 (0.97, 1.33) | 68.6% | < 0.001 | 0.250 | 0.117 | 60.83% | 14.92% |
| Inadequate | 1209 | 9 | 1.38(1.16, 1.65) | 25.4% | 0.218 |  |  |  |  |
| **Incomplete outcome data** | | | | | | | | | |
| Adequate | 5117 | 24 | 1.19 (1.04, 1.37) | 65.6% | < 0.001 | 0.192 | 0.440 | 64.17% | -1.82% |
| Inadequate | 999 | 3 | 1.45 (0.95, 2.19) | 47.5% | 0.149 |  |  |  |  |
| **Selective reporting** | | | | | | | | | |
| Adequate | 6073 | 26 | 1.20 (1.06, 1.37) | 65.0% | < 0.001 | 0.572 | 0.359 | 64.59% | 1.36% |
| Inadequate | 43 | 1 | 2.15 (0.78, 5.92) | / | / |  |  |  |  |
| **Other bias** | | | | | | | | | |
| No | 5183 | 25 | 1.25 (1.09, 1.43) | 63.3% | < 0.001 | -0.326 | 0.188 | 64.73% | -0.86% |
| Yes | 933 | 2 | 0.90 (0.52, 1.57) | 84.6% | 0.011 |  |  |  |  |

The "Blinding of patients" and " Type of control group " variables classified trials in the same subgroups, and therefore had the same meta-regression and subgroup analysis results.(Table 3)

| **Live birth** |  |  |  |  |  |  |  |  |  |
| --- | --- | --- | --- | --- | --- | --- | --- | --- | --- |
| **Characteristic** | **Subgroup analyses** | | | | | **Meta-regression** | | | |
|  | **No. of subjects** | **No. of studies** | **Random-effects RR (95 % CI)** | **Heterogeneity** | | **Coefficient** | ***p*-value** | **I^2^_resid_** | **Adj R^2^** |
|  |  |  |  | **I^2^** | ***P*** |  |  |  |  |
| **Random sequence generation** | | | | | | | | | |
| Adequate | 3911 | 11 | 1.06 (0.89, 1.26) | 62.4% | 0.003 | 0.419 | 0.095 | 55.33% | 35.41% |
| Inadequate | 561 | 4 | 1.66 (1.22, 2.28) | 0% | 0.448 |  |  |  |  |
| **Allocation concealment** | | | | | | | | | |
| Adequate | 3181 | 10 | 1.11 (0.88, 1.39) | 69.8% | < 0.001 | 0.118 | 0.584 | 63.75% | -6.65% |
| Inadequate | 1291 | 5 | 1.22 (0.96, 1.56) | 36.6% | 0.178 |  |  |  |  |
| **Incomplete outcome data** | | | | | | | | | |
| Adequate | 5292 | 26 | 1.16(0.96, 1.40) | 65.9% | < 0.001 | -0.139 | 0.703 | 65.75% | -15.42% |
| Inadequate | 824 | 1 | 1.01(0.75, 1.36) | / | / |  |  |  |  |
| **Other bias** | | | | | | | | | |
| No | 3548 | 13 | 1.21 (1.00, 1.46) | 63.4% | 0.001 | -0.378 | 0.167 | 63.43% | 3.77% |
| Yes | 924 | 2 | 0.83 (0.53, 1.30) | 65.1% | 0.091 |  |  |  |  |

The "Blinding of patients" and " Type of control group " variables classified trials in the same subgroups, and therefore had the same meta-regression and subgroup analysis results.(Table 3)

All the 14 studies which reported LBR were at low risk of bias related to the " Incomplete outcome data " and " Type of control group "
