# Supplementary material for: The effects of acupuncture on pregnancy outcomes of in vitro fertilization: a systematic review and meta-analysis
Source: BMC Complement Altern Med. 2019 Jun 14;19:131. doi: 10.1186/s12906-019-2523-7 (PMC6570865; doi:10.1186/s12906-019-2523-7)
Supplement: Supplementary file 8 — Appendix 1. The reasons for exclusion studies. (DOC 175 kb) [file 12906_2019_2523_MOESM8_ESM.doc]

**Additional file 3: Appendix 1.** **The reasons for exclusion studies.**

Seventy-four RCTs involving acupuncture and IVF were identified. Forty-seven trials were excluded: Magarelli *et al*., Balk *et al.* and Pastuszek *et al*. adopted no random design, Labarta *et al*. and Wang *et al*. were case control studies, and Johnson *et al*. was a case series. Sun *et al*. and Cui *et al*. were duplicate studies, as well as Hong *et al*. and Hong *et al*., the above and Lian *et al*. , Zhou *et al*. all performed an inadequate randomization process in the study design, based on odd and even numbers. Smith *et al*.  and Zheng *et al*.  were protocols and their outcomes were not available. Nine studies used non-invasive acupuncture design, Sun *et al*., Zheng *et al*., Zhang *et al*. , Shuai *et al*. , Zhong *et al*., and Xing *et al*. performed transcutaneous electrical acupoint stimulation as invention, Qu *et al*. , Gay *et al*. and Ng *et al*. adopted auricular acupressure and Streitberger's placebo needles as inventions. Humaidan *et al*. , Kong *et al*. and Wu *et al*. used different stimulation parameters from the intervention groups as controls. Benson *et al*. and Fratterelli *et al*. were duplicate studies with Morin e*t al*., Craig *et al*. 2007 were duplicate study with Craig *et al*. 2014 . We included Morin *et al*. and Craig *et al*. 2014 as they provided more information for our analysis. Zhang *et al*. 2001 , Zhang *et al*. 2002 and Zhang *et al*. 2003 were judged to be derivative publications of the Paulus *et al*. 2002  and Paulus *et al*. 2003 studies, although these article described somewhat different methods and results from each other, as well as Seto *et al*. 2016 and So *et al*. 2009. We included the three latters as they provided more date. Chen *et al*. and Zhang *et al*. 2013 didn't report pregnancy rates, and Gejervall *et al*. only reported the biochemical pregnancy rate, so we excluded these three studies. Udoff *et al*. 2006 and Udoff *et al*. 2014 were duplicate studies, and the data for the exact pregnancy events and totals in acupuncture arm were not available, because the trial used pregnancy rates only. The data on the number of canceled IVF cycles in Li *et al*. was inconsistent, so we excluded this study. Three papers were duplicate studies, they and the other three trials all used ambiguous acupuncture time, this means the quantity of acupuncture stimulus may different between participants, which may result in some unidentified confounders. Quintero *et al*. 2004 was a randomized-control and double-blind trial, and also a crossover pilot study using a needle-like device for the sham acupuncture control.

**References**

1. Magarelli PC, Cridennda DK, Cohen M: **Changes in serum cortisol and prolactin associated with acupuncture during controlled ovarian hyperstimulation in women undergoing in vitro fertilization–embryo transfer treatment**. *Fertility and Sterility* 2009, **92**(6):1870-1879.

2. Balk J, Catov J, Horn B, Gecsi K, Wakim A: **The relationship between perceived stress, acupuncture, and pregnancy rates among IVF patients: a pilot study**. *Complementary therapies in clinical practice* 2010, **16**(3):154-157.

3. Pastuszek E, Liss J, Kulwikowska P, Wisniewska J, Lukaszuk K: **Influence of a cupunctur eonpre gnancyrates in womenundergoinginvitrofertilization**. *Polish Annals of Medicine* 2013, **20**(2):117-123.

4. Labarta E, Privitera L, Bosch E, Ruiz A, Remohí J, Pellicer A: **Effects of acupuncture on reproductive cycle outcome in patients undergoing oocyte donation treatment: A case-Control study**. *Fertility and Sterility* 2010, **94**(4):S173.

5. W W, JH C, JR L, JK C: **A matched controlled study to evaluate the efficacy of acupuncture for improving pregnancy rates following in vitro fertilization-embryo transfer**. *Clinical and experimental obstetrics and gynecology* 2007, **34**(3):137-138.

6. Johnson D: **Acupuncture prior to and at embryo transfer in an assisted conception unit--a case series**. *Acupunct Med* 2006, **24**(1):23-28.

7. Sun W, Cui W, Li J: **Study of the mechanism of electro-acupuncture treatment on PCOS and asthenia of kidney**. *Chinese Journal of Birth Health & Heredity* 2010(01):105-107.

8. Cui W, Li J, Sun W, Wen J: **[Effect of electroacupuncture on oocyte quality and pregnancy for patients with PCOS undergoing in vitro fertilization and embryo transfervitro fertilization and embryo transfer]**. *Zhongguo Zhen Jiu* 2011, **31**(8):687-691.

9. Hong YL, Tan Y, Yin YY, Zou YJ, Guo YH, Nie XW: **Effect of electro-acupuncture on clinical outcomes and ovarian hyperstimulation syndrome in in vitro fertilization and embryo transplantation**. *Zhongguo Zhong xi yi jie he za zhi Zhongguo Zhongxiyi jiehe zazhi = Chinese journal of integrated traditional and Western medicine / Zhongguo Zhong xi yi jie he xue hui, Zhongguo Zhong yi yan jiu yuan zhu ban* 2014, **34**(11):1292-1296.

10. Hong YL: **Inflence of electro-acupuncture on the occurrence of ovarian hyper-stimulation syndrome and outcome of in vitro fertilization and embryo transplantation**. *China Journal of Traditional Chinese Medicine and Pharmacy* 2015, **30**(6):2110-2113.

11. Lian F, Chen C, Xiang S: **Improvement of the oocyte quality with electroacupuncture in infertility patients of kidney deficiency pattern**. *Zhongguo zhen jiu = Chinese acupuncture & moxibustion* 2015, **35**(2):109-113.

12. Zhou L, Xia Y, Ma X, Tang L, Lu J, Tang Q, Wang Y: **Effects of "menstrual cycle-based acupuncture therapy" on IVF-ET in patients with decline in ovarian reserve**. *Zhongguo zhen jiu = Chinese acupuncture & moxibustion* 2016, **36**(1):25-28.

13. Smith CA, de Lacey S, Chapman M, Ratcliffe J, Norman RJ, Johnson N, Sacks G, Lyttleton J, Boothroyd C: **Acupuncture to improve live birth rates for women undergoing in vitro fertilization: a protocol for a randomized controlled trial**. *Trials* 2012, **13**:60.

14. Zheng CH, Zhang J, Wu J, Zhang MM: **The effect of transcutaneous electrical acupoint stimulation on pregnancy rates in women undergoing in vitro fertilization: a study protocol for a randomized controlled trial**. *Trials* 2014, **15**:162.

15. Sun W, Tian L, Feng X, Zhang R, Wang S, Li R, Kong R, Zhang X, Zhou L, Yang S *et al*: **Pain relief during oocyte retrieval by transcutaneous electrical acupoint stimulation: A single blind randomized controlled multi-centered trial**. *Human* 2017, **32**:i485-i486.

16. Zheng Y, Feng X, Mi H, Yao Y, Zhao Y, Li J, Jiao J, Gong A, Sun W, Deng X: **Effects of transcutaneous electrical acupoint stimulation on ovarian reserve of patients with diminished ovarian reserve in in vitro fertilization and embryo transfer cycles**. *The journal of obstetrics and gynaecology research* 2015, **41**(12):1905-1911.

17. Zhang R, Feng XJ, Guan Q, Cui W, Zheng Y, Sun W, Han JS: **Increase of success rate for women undergoing embryo transfer by transcutaneous electrical acupoint stimulation: a prospective randomized placebo-controlled study**. *Fertil Steril* 2011, **96**(4):912-916.

18. Shuai Z, Lian F, Li P, Yang W: **Effect of transcutaneous electrical acupuncture point stimulation on endometrial receptivity in women undergoing frozen-thawed embryo transfer: a single-blind prospective randomised controlled trial**. *Acupunct Med* 2015, **33**(1):9-15.

19. Zhong J, Zhang L: **[Transcutaneous electrical acupoint stimulation for pregnancy of in vitro fertilization-embryo transfer]**. *Zhongguo Zhen Jiu* 2017, **37**(3):253-255.

20. Xing LF, Wu Y, He YJ, Wang FF, Zhu YH, Ma CM, Yan YY, Sun SJ, Pan JX, Robinson N *et al*: **Transcutaneous electrical acupoint stimulation alleviates the anxiety levels of IVF: A prospective, randomized and controlled study**. *European Journal of Integrative Medicine* 2018, **20**:126-130.

21. Ng EHY, So EWS, Li RHW, Yeung WSB, Ho PC: **Effect of non-invasive acupuncture on the pregnancy outcome of IVF treatmentóa randomized controlled study**. *Human Reproduction* 2011, **26**:i70.

22. Gay C, Cros A, Berbis J, Bretelle F, Perrin J, Courbiere B: **Evaluation of "energy Resonance by Cutaneous Stimulation" among Women Treated by in Vitro Fertilization**. *Journal of Alternative and Complementary Medicine* 2018, **24**(6):578-583.

23. Humaidan P, Brock K, Bungum L, Stener-Victorin E: **Pain relief during oocyte retrieval--exploring the role of different frequencies of electro-acupuncture**. *Reprod Biomed Online* 2006, **13**(1):120-125.

24. Kong S, Hughes A: **Acupuncture as an adjunct to in vitro fertilization: A randomized trial**. *Medical Acupuncture* 2009, **21**(3):179-182.

25. Wu R, Wang Y: **Application time-point and effect observation of fire needling therapy in IVF-ET**. *Zhongguo Zhen Jiu* 2017, **37**(5):498-502.

26. Benson MR, Elkind-Hirsch KE, Theall A, Fong K, Hogan RB, Scott RT: **Impact of acupuncture before and after embryo transfer on the outcome of in vitro fertilization cycles: A prospective single blind randomized study**. *Fertility And Sterility* 2006, **86**:S135-S135.

27. Fratterelli JL, Leondires MR, Fong K, Theall A, Locatelli S, Scott RT: **Laser acupuncture before and after embryo transfer improves ART delivery rates: results of a prospective randomized double-blinded placebo controlled five-armed trial involving 1000 patients**. *Fertility and Sterility* 2008, **90**(0):S105.

28. Morin SJ, Frattarelli JL, Franasiak JM, Juneau CR, Scott RT: **Laser Acupuncture before and after Embryo Transfer Improves in Vitro Fertilization Outcomes: A Four-Armed Randomized Controlled Trial**. *Medical* 2017, **29**(2):56-65.

29. Craig LB, Criniti AR, Hansen KR, Marshall LA, Soules MR: **Acupuncture lowers pregnancy rates when performed before and after embryo transfer**. In: *Fertility and sterility.* vol. 88 Suppl 1; 2007: 40, Abstract no: 106.

30. Craig LB, Rubin LE, Peck JD, Anderson M, Marshall LA, Soules MR: **Acupuncture performed before and after embryo transfer: a randomized controlled trial**. *The Journal of reproductive medicine* 2014, **59**(5-6):313-320.

31. Zhang M, Paulus W, Strehler E, Jelinkova L, Sterzik K: **Increase of pregnancy rate in assisted reproduction therapy by acupuncture**. In: *Fertility and sterility.* vol. 76; 2001: S75.

32. Zhang M, Huang G, Lu F, El-Danasouri I, Sterzik K: **Effect of acupuncture on rate of pregnancy among women undergoing embryo transfer**. *Chinese acupuncture & moxibustion* 2002, **22**(8):507-509.

33. Zhang M, Huang G, Lu F, El-Danasouri I, Sterzik K: **Effect of acupuncture on rate of pregnancy among women undergoing embryo transfer: a prospective randomized placebo controlled trial.** *Chinese acupuncture & moxibustion* 2003, **23**(1):3-5.

34. Paulus WE, Zhang M, Strehler E, El-Danasouri I, Sterzik K: **Influence of acupuncture on the pregnancy rate in patients who undergo assisted reproduction therapy**. *Fertil Steril* 2002, **77**(4):721-724.

35. Paulus WE, Zhang M, Strehler E, Seybold B, Sterzik K: **Placebo-controlled trail of acupuncture effects in assisted reproduction therapy**. *Human Reproduction* 2003, **18**:18-19.

36. Seto MTY, Cheung KW, Lo TK, Ng EHY: **A retrospective review of pregnancy outcomes of women randomised to receive real versus placebo acupuncture on the day of embryo transfer**. *BJOG: An International Journal of Obstetrics and Gynaecology* 2016, **123**:114-115.

37. So EWS, Ng EHY, Wong YY, Lau EYL, Yeung WSB, Ho PC: **A randomized double blind comparison of real and placebo acupuncture in IVF treatment**. *Human Reproduction* 2009, **24**(2):341-348.

38. Chen QQ, Wei QL, Zhang XH: **[Effects of electroacupuncture on supplementary analgesia and improvement of adverse reactions induced by dolantin in oocyte retrieval]**. *Zhongguo Zhen Jiu* 2012, **32**(12):1113-1116.

39. Zhang J, Wang X, Lu R: **Analgesic effect of acupuncture at hegu (LI 4) on transvaginal oocyte retrieval with ultrasonography**. *Journal of traditional Chinese medicine = Chung i tsa chih ying wen pan* 2013, **33**(3):294-297.

40. Gejervall AL, Stener-Victorin E, Möller A, Janson PO, Werner C, Bergh C: **Electro-acupuncture versus conventional analgesia: a comparison of pain levels during oocyte aspiration and patients' experiences of well-being after surgery**. In: *Human reproduction (Oxford, England).* vol. 20; 2005: 728-735.

41. Udoff LC, Zhang G, Patwardhan S, Wei Z, McClamrock HD: **P-45: The effect of acupuncture on outcomes in in-vitro fertilization (IVF)**. *Fertility and Sterility* 2006, **86**(3, Supplement):S145.

42. Udoff LC, McClamrock HD, Chen K, Zhang G: **The effect of acupuncture on pregnancy out comes in in-vitro fertilization (IVF) : Arandomized controlled trial**. *Fertility and Sterility* 2014, **102**(3):e333.

43. Li J, Cui W, Sun W: **Effects of electroacupuncture on in vitro fertilization - embryo tranfer(IVF-ET) if patients with polycystic ovary syndrome**. *Ch in J Hum Sex* 2009(07):28-30.

44. Li J, Cui W, Sun W, Zhang QY, Guan Q: **Effect of electro-acupuncture on the spindle and oocytes quality in patients with PCOS**. *Chinese journal of integrated traditional and Western medicine* 2015, **35**(3):304-309.

45. Li J, Cui W, Sun W, Wen J: **Effect of electroacupuncture on egg quality and tumor necrosis factor -α of patients with polycystic ovarian syndrome**. *World Journal of Acupuncture - Moxibustion* 2014, **24**(3):9-15.

46. Yang BZ, Cui W, Li J: **Effects of electroacupuncture intervention on changes of quality of ovum and pregnancy out- come in patients with polycystic ovarian syndrome**. *Zhen ci yan jiu = Acupuncture research* 2015, **40**(2):151-156.

47. Chen J, Liu LL, Cui W, Sun W: **Effects of electroacupuncture on in vitro fertilization-embryo transfer (IVF-ET) of patients with poor ovarian response**. *Zhongguo Zhen Jiu* 2009, **29**(10):775-779.

48. Cui W, Sun W, Li J, Wen J, Feng XJ: **Influence of using electro-acupuncture during COH on the pregnancy outcome in patients undergoing in-vitro-fertilization and embryo transfer**. *Shandong Medical* 2012, **52**(37):7-10.

49. Cui W, Sun W, Liu LL, Wen J: **Study of the effect of electro-acupuncture treatment on the patients undergomg in vitro-fertilization and embryo transfer**. *Chin Matern Child Health* 2007, **22**:3403–3405.

50. Chen Q, Hau C: **Impacts on pregnancy outcome treated with acupuncture and moxibustion in IVF-ET patients**. *Zhongguo zhen jiu = Chinese acupuncture & moxibustion* 2015, **35**(4):313-317.

51. Wang XM, Jing FQ, Wang C, Tian LJ, Liu H, Fang LN, Zhou W, Lei H, Zhou Y: **The influence for success rate of warm acupuncture for tube baby of infertility patients with kidney yang deficiency: a randomized controlled trial**. *Chinese acupuncture & moxibustion* 2016(09):906-910.

52. Quintero R: **A randomized, controlled, double-blind cross-over study evaluating acupuncture as an adjunct to IVF**. *Fertil Steril* 2004, **81**:S11-12.
